# Refinement of evolutionary medicine predictions based on clinical evidence for the manifestations of Mendelian diseases

**DOI:** 10.1038/s41598-019-54976-4

**Published:** 2019-12-09

**Authors:** Daniela Šimčíková, Petr Heneberg

**Affiliations:** Charles University, Third Faculty of Medicine, Prague, Czech Republic

**Keywords:** Disease genetics, Endocrine system and metabolic diseases, Molecular medicine

## Abstract

Prediction methods have become an integral part of biomedical and biotechnological research. However, their clinical interpretations are largely based on biochemical or molecular data, but not clinical data. Here, we focus on improving the reliability and clinical applicability of prediction algorithms. We assembled and curated two large non-overlapping large databases of clinical phenotypes. These phenotypes were caused by missense variations in 44 and 63 genes associated with Mendelian diseases. We used these databases to establish and validate the model, allowing us to improve the predictions obtained from EVmutation, SNAP2 and PoPMuSiC 2.1. The predictions of clinical effects suffered from a lack of specificity, which appears to be the common constraint of all recently used prediction methods, although predictions mediated by these methods are associated with nearly absolute sensitivity. We introduced evidence-based tailoring of the default settings of the prediction methods; this tailoring substantially improved the prediction outcomes. Additionally, the comparisons of the clinically observed and theoretical variations led to the identification of large previously unreported pools of variations that were under negative selection during molecular evolution. The evolutionary variation analysis approach described here is the first to enable the highly specific identification of likely disease-causing missense variations that have not yet been associated with any clinical phenotype.

## Introduction

Computational prediction approaches are an integral part of biomedical and biotechnological research. The prediction algorithms have great potential in precision medicine, particularly with their recent applications in filtering the exome sequencing outcomes for facilitating diagnoses of rare, hardly classifiable, or puzzling disorders suspected of having a genetic origin^1,2^. The vast majority of coding variations are rare and limited functional data are available^3,4^. This limited availability of evidence-based information is the main argument for the use of prediction algorithms. The prediction algorithms clearly do not outperform evidence-based data in determining the effects of individual variations. However, they allow researchers and clinical geneticists to extrapolate of current knowledge to genes or variations with as yet unknown or uncertain phenotypes. Among the most important modes of use of the prediction algorithms is the assessment of the likely pathogenicity of variations that are discovered *de novo* during exome sequencing studies and in other next-generation sequencing data. Improvements in methods for predicting the pathogenicity of rare coding variations are needed^5^. Although rare coding variations are often neglected, approximately 100–400 of these variations are present in the genome of each human^3,4^ and many have been shown to cause inherited diseases^6,7^. As we have shown in the pilot study that focused on the glucokinase (GCK), the potential to substantially improve outcomes of already available computational prediction approaches exists when matching them with evidence-based functional data related to clinically reported and/or experimentally analyzed variations in the respective gene^8^.

Most prediction methods assume the *de novo* protein structure and function based on the knowledge of structural features of wild-type proteins and amino acid sequences and their evolutionary conservation^9–11^. Similar approaches have been used to decipher the effects of variations in non-coding sequences^12^. Some approaches, such as PoPMuSiC 2.1^13^, also consider protein thermostability in their estimations^14–16^. The prediction methods may be supervised and thus trained and tested on a properly assembled dataset with reliable annotations^15,17^. Alternatively, they may be designed as autonomous unsupervised methods, which have better generalization properties and are able to recognize potentially novel types of omics elements^12,14,17^, but are not resistant to errors incorporated during their development. Most of the prediction methods are based on the evolution-based concept^12^. However, the evolutionary sequence information poorly covers the additive roles of environmental factors, and the building and interpretation of multiple sequence alignments (MSAs) is still unable to be fully automated^18,19^. Many prediction approaches integrate multiple biophysical characteristics; a classical example of these approaches is SNAP2^20^. Another strategy that increases the specificity and selectivity is the use of consensus classifiers, such as REVEL^5^, which integrate outcomes of multiple prediction algorithms to eliminate randomly occurring false-positive responses of the individual algorithms. Recently, the traditional approaches were outperformed by an unsupervised prediction method termed EVmutation^14^, which considers epistasis and thus reflects dependencies between positions^21,22^. When the epistasis is reflected in the inference and subsequent use of MSAs, certain variations are labeled as non-acceptable, although they are frequently observed in other positions within the sequence^14,23^, highlighting the need to incorporate the epistatic approach in individual computational algorithms and consensus classifiers.

In the present study, we hypothesized that the reliability of prediction methods would be improved by switching from *ad hoc* to evidence-based thresholds and provide a proof of concept by modelling and validating this approach for genes associated with Mendelian diseases. We focus on the differences between clinically observed missense variations that are or are not associated with Mendelian diseases and show that the use of evidence-based tailored thresholds substantially improves the prediction of causative disease-associated missense variations (DAVs) among newly identified variations in the course of genomic and proteomic screens.

## Materials and Methods

**Figure 1:**
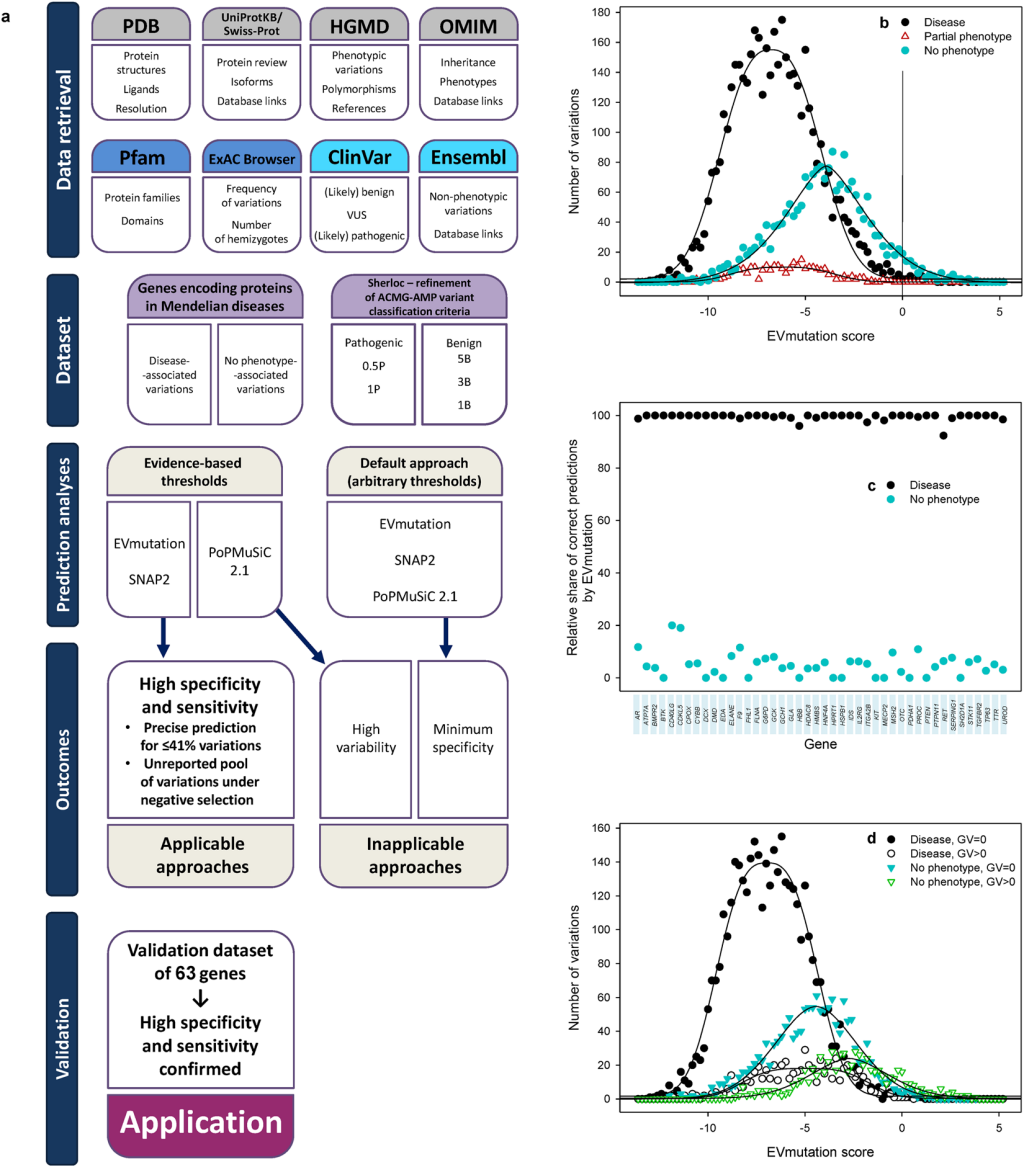
The efficiency of the EVmutation method in predicting the effects of missense variations with known clinical Mendelian disease-associated phenotypes. (**a**) Flowchart showing the sources and approaches used for data retrieval, the construction of datasets and subsequent analyses. The selection of analyzed genes associated with Mendelian diseases was based on combined information retrieved from the Human Gene Mutation Database (HGMD), UniProtKB/Swiss-Prot, Protein Data Bank (PDB) and Online Mendelian Inheritance in Man (OMIM). Information about disease associations and no-phenotype associations of clinically observed variations was retrieved from the ClinVar database and the Ensembl browser. Additional information about proteins (domains) and variations (frequency) was obtained from the Pfam database and the Exome Aggregation Consortium (ExAC) browser, respectively. A vertical line indicates the arbitrary threshold for variations with an effect. (**b**) The distribution of numerical EVmutation scores calculated for missense variations with known clinical phenotypes. (**c**) The relative percentage of correct predictions of disease and no clinical phenotypes using EVmutation scores calculated for the 44 analyzed proteins. (**d**) The distribution of numerical EVmutation scores calculated for disease-associated and no phenotype-associated missense variations with known clinical phenotypes in 44 proteins that cause Mendelian diseases sorted according to the evolutionary conservation of affected amino acids in mammals. Conserved amino acids (GV = 0) were conserved in all ten examined mammalian orthologs. Variable amino acids (GV > 0) were not conserved in at least one of the ten examined mammalian orthologs of the respective protein.

We assembled two curated databases of missense variations in genes encoding proteins associated with Mendelian diseases to establish and validate the model (Fig. 1a). When establishing the model, we recognized three categories of variations: (1) “DAVs” represented variations with available evidence of an association with Mendelian diseases. (2) “Partial phenotype-associated” variations were reported to be associated with partial (incompletely manifesting) phenotypes of the same Mendelian diseases. And (3) “No phenotype-associated” variations (NPAVs) were variations with conclusive evidence of the absence of any clinical phenotype associated with their carriers. We predicted the effects of variations using EVmutation^14^ based on a specific epistatic model, SNAP2^20^, which is based on multiple biophysical characteristics, and PoPMuSiC 2.1^13^ that predicts protein thermostability.

In addition to the clinically observed variations, we calculated and analyzed the predictions for theoretical variations, i.e., variations that have not been clinically observed. We sorted the variations according to (a) their localization within/outside protein domains, (b) the presence and class of enzymatic activity of the protein, (c) the number of nucleotide changes needed to obtain the variation of interest, and (d) the American College of Medical Genetics and Genomics (ACMG) classification criteria^24^.

### Selection of genes to establish the model

We selected genes encoding proteins associated with Mendelian diseases according to the availability of a protein structure, inheritance of diseases, and sufficient numbers of clinically observed missense variations (at least nine missense DAVs and at least six missense NPAVs in a region for which the protein structure was available). We retrieved data from the Online Mendelian Inheritance in Man (OMIM; https://omim.org/), UniProtKB/Swiss-Prot (http://www.uniprot.org/), Protein Data Bank (PDB; https://www.rcsb.org/ and Human Gene Mutation Database (HGMD; http://www.hgmd.cf.ac.uk). We obtained the evidence for the presence of NPAVs from the ClinVar (https://www.ncbi.nlm.nih.gov/clinvar/) and Ensembl (http://www.ensembl.org/) databases. We completed information with frequencies of variations and protein domains obtained from the Exome Aggregation Consortium browser (ExAC; http://exac.broadinstitute.org/) and the Pfam (http://pfam.xfam.org/) database, respectively.

We verified all ambiguous data in the primary literature sources. If we observed conflicting evidence or if conclusive evidence was not available, we removed the variations from the analyses. The factors that led to the removal of variations from the analyzed datasets are listed below. (1) The evidence for only non-Mendelian diseases (e.g., Parkinson disease) was manifested in the carriers of the variation. (2) The variations were listed as benign or likely benign in ClinVar, with high frequencies (*f* > 8) in ExAC, and thus were classified as 1B or higher according to the ACMG criteria for high-quality and abundant data^25^. (3) The variations were listed as “DM?” in the HGMD database. These variations denote “a probable/possible pathological mutation, reported to be pathogenic in the corresponding report, but for which (1) the author has indicated that there may be some degree of uncertainty; (2) the HGMD curators believe greater interpretational caution is warranted; or (3) subsequent evidence has appeared in the literature which has called the putatively deleterious nature of the variant into question”^26^. (4) Variations for which a disagreement occurred between HGMD (classified as “DM”) and ClinVar (classified as “benign” or “likely benign”).

We used the key provided in Table 1 to assign of the clinically observed variations. We selected all clinically observed variations, which we used to set the thresholds, using the key described above. Additionally, we included the GCK variations resulting from the systematic literature review provided in 2017 by Šimčíková *et al*.^8^ We classified nine variations as NPAVs based on the recent literature^27–32^. We included the hemoglobin variations, which were classified as likely non-phenotypic in the HGMD database, in the NPAVs.

**Table 1:** The key used to assign of the clinically observed variations.

1a)	In HGMD, the variation is absent.	2
1b)	In HGMD, the variation is present, but causes “no phenotype” according to dbSNP.	NO PHEN
1c)	In HGMD, the variation is present and is defined as a “disease-causing mutation”.	4
1d)	In HGMD, the variation is present but has definitions other than those listed in 1b) and 1c)	2
2a)	In ClinVar, the variation is present and defined as “benign”, “likely benign” or “variants of uncertain significance” (VUSs).	NO PHEN
2b)	In ClinVar, the variation is absent or present, with definitions other than those listed in 2a).	3
3a)	In Ensembl, the variation is present but has no associated phenotype.	NO PHEN
3b)	In Ensembl, the variation is present and associated with a phenotype.	5
4a)	In ClinVar, the variation is present and defined as “benign” or “likely benign”.	EXCL
4b)	In ClinVar, the variation is present but not defined as “benign” or “likely benign”.	5
5a)	In HGMD, all variations classified as “disease-causing mutations” within the respective gene are associated with a single disease or syndrome with a Mendelian inheritance pattern.	DIS
5b)	In HGMD, the variations classified as “disease-causing mutations” within the respective gene are associated with two diseases with a Mendelian inheritance pattern, one caused by the activating and the other by inactivating variations (e.g., erythrocytosis *vs* anemia).	DIS
5c)	In HGMD, the variations classified as “disease-causing mutations” within the respective gene are associated with two diseases with a Mendelian inheritance pattern, both of which are caused by variations exerting similar effects with a different intensity (e.g., Menkes syndrome *vs* occipital horn syndrome or Duchenne *vs* Becker muscular dystrophy); variations cause a complete phenotype.	DIS
5d)	In HGMD, the variations classified as “disease-causing mutations” within the respective gene are associated with two diseases with a Mendelian inheritance pattern, both of which are caused by variations exerting similar effects with a different intensity (e.g., Menkes syndrome *vs* occipital horn syndrome or Duchenne *vs* Becker muscular dystrophy); variations cause the less pathological phenotype.	PART

Abbreviations used: DIS – disease-associated; PART – partial phenotype-associated; NO PHEN – no phenotype-associated; EXCL – excluded ambiguous data.

The variations classified in ClinVar as VUS (n = 404) were subjected to the analysis using EVmutation, and SNAP2 scores shifted slightly but significantly towards their pathogenicity compared to the variations classified as benign or likely benign (n = 1589): EVmutation mean ± SD −4.21 ± 2.54 *vs* −3.84 ± 2.38, *t*-test *p = *0.003; SNAP2 mean ± SD −0.9 ± 57.34 *vs* −12.57 ± 55.85, *t*-test *p < *0.001. Based on these calculations, we excluded the hemoglobin variations that were classified as likely non-phenotypic in HGMD (n = 100). These variations received EVmutation scores, but not SNAP2 scores, similar to VUS (EVmutation mean ± SD −4.26 ± 2.25, *t*-test *vs* VUS *p* > 0.05; SNAP2 mean ± SD 15.58 ± 42.69, *t*-test *vs* VUS *p = *0.003).

All variations included in the dataset we used to establish the model were classified according to the ACMG criteria^24^, differentiating between those classified as benign (1B, 3B and 5B) and pathogenic (0.5P and 1P). The frequencies of the variations according to the ACMG classification are provided in Table S10.

We retrieved clinical information on 7178 missense variations (Fig. 1a) located within the coding sequences of 44 genes that, if mutated, cause Mendelian diseases. The following genes were included in the dataset we used to validate the model: *AR*, *ATP7A*, *BMPR2*, *BTK*, *CD40LG*, *CDKL5*, *CPOX*, *CYBB*, *DCX*, *DMD*, *EDA*, *ELANE*, *F9*, *FHL1*, *FLNA*, *G6PD*, *GCK*, *GCH1*, *GLA*, *HBB*, *HDAC8*, *HMBS*, *HNF4A*, *HPRT1*, *HSPB1*, *IDS*, *IL2RG*, *ITGA2B*, *KIT*, *MECP2*, *MSH2*, *OTC*, *PDHA1*, *PROC*, *PTEN*, *PTPN11*, *RET*, *SERPING1*, *SH2D1A*, *STK11*, *TGFBR2*, *TP63*, *TTR* and *UROD*. All the analyzed missense variations were limited to those parts of the genes for which structural information was available. We designated 4546 variations as “DAVs”, because the evidence for their associations with Mendelian diseases was available. We designated another 291 variations as “partial phenotype-associated”, because the evidence for their association with partial (incompletely manifesting) phenotypes of the same Mendelian diseases was available. We designated 2093 missense variations as “NPAVs”, because conclusive evidence of the absence of any clinical phenotype associated with their carriers was available. We removed 248 (3.5%) missense variations from the analyses due to inconsistent, insufficient or anomalous data on the phenotypes reportedly associated with these variations. Data reliability in databases appears to be a challenge to the construction of the dataset. Standardized forms of annotations do not currently exist. Additionally, submission processes differ among the databases, ranging from individual to bulk submissions, and are rarely checked for consistency with previously published peer-reviewed studies^31^. Therefore, the construction of the comprehensive dataset also prevented or considerably decreased the risk of biases that might arise from errors of omission and commission in databases.

### Selection of genes to validate the model

We established the validation dataset consisting of 1723 variations in 63 additional genes associated with autosomal dominant or autosomal recessive diseases to validate the newly reported approach on an independent set of proteins that are associated with Mendelian diseases (Table S8). These 63 genes were not included in the dataset that was used to establish the model. We populated the dataset based on the classifications of variations retrieved from ClinVar. We also verified the allele counts in the ExAC browser, but this information was only available for a limited number of variations in this dataset. Thus, this information was not used in the analyses. The genes included in the dataset that was used to validate the model were: *AARS*, *ABCC6*, *ALDH18A1*, *ARSB*, *AVP*, *CASR*, *CFTR*, *CLCN1*, *CLCN7*, *COL7A1*, *DNM2*, *DSP*, *DYNC1H1*, *ELOVL4*, *FBN1*, *FGF23*, *FGFR3*, *GALNS*, *GBA*, *GJB2*, *GJA3*, *GLB1*, *GNE*, *GUCY2D*, *GUSB*, *HEXA*, *HGSNAT*, *IMPDH1*, *KCNA1*, *LMNA*, *LMNB1*, *LRP5*, *MARS*, *MPZ*, *MYH14*, *MYH3*, *MYH7*, *MYH9*, *MYO6*, *NAGLU*, *NOTCH3*, *NR3C2*, *OPA1*, *PGFRB*, *PKD1*, *PKD2*, *POLG2*, *PRKCG*, *PRPF8*, *RAF1*, *RYR1*, *SGSH*, *SLC4A1*, *SMPD1*, *SOS1*, *SOS2*, *SPAST*, *STAT1*, *STAT3*, *TECTA*, *TERT*, *VCP* and *YARS*. The dataset was composed of the following numbers of variations: 33 benign, 53 benign/likely benign variations, 58 likely benign variations, 475 likely pathogenic variations, 104 likely pathogenic/pathogenic variations and 1000 pathogenic variations (Table S8).

### Prediction analyses

For all selected proteins, we employed three methods with distinct approaches and bases. First, we used the unsupervised epistatic model EVmutation^14^ with the arbitrary threshold set to zero. Second, we used the supervised method SNAP2^20^, which is based on multiple biophysical characteristics and trained on annotated databases of clinically observed and/or experimentally tested variations from annotated databases (OMIM, PMD and Swiss-Prot). Third, we used PoPMuSiC 2.1^13^, which predicts protein thermostability. The arbitrary threshold of the EVmutation method was set to zero based on the claim by Hopf *et al*.^14^ that “values of ∆E above 0 correspond to more probable mutant sequences (putatively beneficial), values below 0 to less probable mutant sequences (putatively deleterious) and values equal to 0 to equally probable sequences (putatively neutral).” Thus, the arbitrary threshold allowed us to differentiate between the “putatively deleterious” and “putatively beneficial” mutations. Based on these criteria, the variation effect scores were also set to zero for all examined wil-type protein sequences in the protein matrices that were precomputed by Hopf *et al*.^14^ (available at https://marks.hms.harvard.edu/evmutation/, accessed March 8, 2018). Due to the nature of the EVmutation method, almost no “putatively neutral” variations with a zero EVmutation score were observed, except for the wild-type alleles. Hopf *et al*. applied these settings to changes occurring at the protein level, but predictions of the changes at the level of the whole organism are more challenging.

We used the pre-computed predictions from EVmutation that were listed according to the UniProtKB/Swiss-Prot accession numbers. We computed the predicted effects of amino acid changes identified using SNAP2 according to the NCBI code belonging to relevant protein isoforms. We selected the protein structures with a resolution lower than 2.7 Å (except *GCH1* and *PROC*) and used their PDB codes in the prediction computations employing PoPMuSiC 2.1. In addition to the clinically confirmed variations, we calculated and analyzed the predictions for theoretical variations, i.e., variations that were not clinically observed. We performed these calculations for the protein regions identical to those, we used to analyze the clinically observed variations. We sorted the variations according to a) their localization within/outside of protein domains, b) the presence and class of enzymatic activity of the protein, and c) the number of nucleotide changes needed to obtain the variation of interest. When sorting the variations according to the latter criterion, we split theoretical variations into impossible (157,639 variations) and possible variations (63,698 variations) according to the method reported by Bromberg *et al*.^15^. They defined “impossible” amino acid variations as those that require a change of two or three nucleotides in the codon, whereas “possible” variations were defined as amino acids variations that require a change in only a single nucleotide.

### GV approach

Many variations that were previously associated with Mendelian diseases have been re-assessed and re-classified as VUSs^32–34^. In the present study, we limited the MSAs based on the paradigm of the VUS^25^ classification, which differentiates VUSs from likely benign variations by analyzing their conservation in other mammalian species. According to multiple indices, the predictions of the effects of the analyzed variations may be improved by implementing MSA analyses. The MSA analyses assume that variations identified in related species are likely neutral (non-pathogenic), whereas variations identified in conserved parts of the amino acid sequence are likely pathogenic. A consensus regarding the inclusion criteria for the analyzed sequences has not been reached. Some authors compare the sequences of all proteins in the respective protein family, while others limit the analyzed sequences to those that are similar to human sequences^33,34^.

We used the GV approach to analyze the MSAs of amino acid sequences of the examined human proteins and their mammalian orthologs^35^. The GV approach quantifies the variability in each tested amino acid based on the MSA provided. This approach allowed us to classify the variations into those with GV scores of zero (conserved among mammals) and those with higher GV scores (with at least two sequence variations present in the analyzed MSAs). We assembled the MSAs by implementing the paradigm associated with variants of uncertain significance (VUS), which claims that the variations are considered VUSs if an amino acid residue that is conserved in the corresponding protein in other mammals is altered^25^. Thus, for each analyzed protein, we prepared the MSA that contained amino acid sequences of ten mammalian orthologs of the respective gene. Typically, we included a dominant human isoform of the respective protein and complemented it with the corresponding isoform reported from two species of primates (Primates) and one sequence each from carnivores (Carnivora), bats (Chiroptera), rodents (Rodentia), even-toed ungulates or cetaceans (Cetartiodactyla) and insectivorous mammals (Eulipotyphla, which is still listed as Insectivora in the NCBI Nucleotide database). The remaining two orthologs were both represented by marsupials (Metatheria) or by one marsupial and one monotreme (Monotremata), avoiding monotreme sequences when high-quality reads were not available in the NCBI GenBank database. We retrieved all sequences from the NCBI GenBank database between May 30 and June 4, 2017.

Additionally, we tested two representative genes, *AR* and *PTEN*, to determine whether the addition of more evolutionarily distant sequences and the resulting increase in variability led to an improved correspondence of GV scores with disease associations of analyzed variations. We used the maximum likelihood method to estimate evolutionary divergence in amino acid sequences predicted to be encoded by *AR* and *PTEN* among selected taxonomic groups. For AR, we tested 29 amino acid sequences of AR orthologs, including the orthologs from ten mammalian species, as specified above. The more evolutionarily distant orthologs included sequences from Testudines (three species), Amphibia (three species), Crocodylia (two species), Squamata (four species), Aves (three species), Euteleostomi (three species) and Chondrichthyes (one species). The NCBI Blast search did not retrieve orthologs that would be homologous with AR from more evolutionarily distant species. The PTEN protein is more evolutionarily conserved, which allowed us to include more distant taxa. The resulting dataset comprised 31 orthologs, ten of which were from the mammalian species listed above, and others consisted of orthologs from the following taxa: Aves (three species), Squamata (three species), Archelosauria (three species), Teleostei (three species), Chondrichthyes, Coelacanthiformes, Amphibia, Brachipoda, Gastropoda, Mollusca, Echinozoa, Arachnida and Insecta (one species each). We retrieved these sequences from the NCBI GenBank database between October 8 and October 14, 2017. We aligned the amino acid sequences using ClustalW (gap opening penalty of 5 and gap extension penalty of 0.1 for pairwise alignments, gap extension penalty of 0.2 for multiple alignments, and gap separation distance of 4). We manually corrected the alignments for any inconsistencies and replaced shorter sequences with more appropriate sequences. We used only sequences of identical lengths for further analyses. We used the resulting MSAs to calculate the GV scores. For the AR and PTEN alignments, we performed maximum likelihood fits of the 48 amino acid substitution models, excluding positions containing gaps. For each model, we calculated the Bayesian information criterion, corrected Akaike information criterion and maximum likelihood values. For AR, we analyzed 29 sequences with 380 positions in the final dataset. For PTEN, we analyzed 31 sequences with 342 positions in the final dataset. We used best-fit models for the subsequent phylogenetic analyses and evolutionary divergence calculations. When building the trees, we constructed the initial tree using a neighbor-joining algorithm. We built the trees based on both AR and PTEN sequences using the Jones-Taylor-Thornton model. We modeled the non-uniformity of evolutionary rates among sites using a discrete Gamma distribution (+G) with five rate categories. We applied a bootstrapping procedure with 1,000 replicates. We used the maximum likelihood method to estimate evolutionary divergence in the amino acid sequences of AR and PTEN orthologs among selected taxonomic groups. We calculated the number of base differences per site by averaging all sequence pairs between groups (distance) ± SE and employed a bootstrapping procedure with 1,000 replicates. The models used to estimate inter- and intrasite evolutionary divergence were identical to the models used to construct the respective trees.

### Revel

We calculated the sensitivity and specificity of the predictions retrieved from REVEL to test whether the issue of low specificity is associated with the outcomes of individual computational algorithms or whether it also affects the data obtained using state-of-the-art consensus classifiers^5^. We used REVEL to test a subset of 21 genes from the dataset that was used to establish the model: *GCK*, *AR*, *PTEN*, *CYBB*, *HNF4A*, *HBB*, *MECP2*, *HDAC8*, *RET*, *PTPN11*, *HPRT1*, *CD40LG*, *CDKL5*, *CPOX*, *DCX*, *DMD*, *EDA*, *UROD*, *TTR*, *FLNA* and *HSPB1*. We provided REVEL scores for 2721 variations, of which 1570 were DAVs, 241 manifested partial phenotypes, and 910 were NPAVs. For the aforementioned genes, we tested the identical set of variations as used to establish the model, except for PTEN p.P103Q, PTEN p.A137F, and four GCK variations, representing amino acid substitutions caused by substitutions of two or three nucleotides. We obtained the REVEL scores from the pre-computed database of REVEL scores that are available for all missense variations retrieved from dbNSFP v2.7, as provided by the authors of REVEL^5^.

### Statistical analyses

We calculated the evidence-based thresholds as medians ± 2 × SD, which should encompass approximately 95% of the pool of variations used to calculate the threshold. We calculated two types of these thresholds. The sensitivity threshold (true positive rate) was calculated based on the 95% chance of confirming the association of a tested theoretical variation with the respective disease based on the distribution of prediction scores for known DAVs. The specificity threshold (true negative rate) was calculated based on the 95% chance of confirming the absence of an association of a tested theoretical variation with the respective disease based on the distribution of prediction scores for known NPAVs.

We calculated the weighted means of the scores resulting from the tested prediction methods by assigning each predictor a weight ranging from −100 to +100, where 0 was a threshold and 100 was the maximum value observed within the respective dataset (EVmutation range from −12.933 to 3.8104, SNAP2 range from −98 to 99, and PoPMuSiC 2.1 range from −1.90 to 5.64), and by averaging the values obtained from each of the prediction methods.

We tested the differences between predictions between DAVs and NPAVs, and for domain-associated and other amino acids using a one-tailed *t*-test. Differences in the numbers of DAVs and NPAVs in individual domains were determined using one-tailed *t*-tests with Bonferroni’s correction. We tested the differences between variations associated with particular classes of enzymes and proteins without enzymatic functions, and between categories of possible and impossible theoretical variations using the Kruskal-Wallis one-way ANOVA on ranks with Dunn’s post-tests (the Kolmogorov-Smirnov normality test yielded *p* > 0.05 for each comparison). We analyzed the difference in the frequency of DAVs and NPAVs among possible and impossible theoretical variations using the χ^2^ test, with the number of possible variations normalized to the number of impossible variations. We assessed the differences between DAVs (including multiple phenotypes alone), partial phenotype-associated and NPAVs using the Kolmogorov-Smirnov normality test followed by one-way ANOVA with Tukey’s post-tests or Kruskal-Wallis one-way ANOVA on ranks with Dunn’s post-tests when the normality tests failed. We did not evaluate phenotypes with less than five associated variations. The data are shown as means ± SD, unless indicated otherwise. We performed all calculations using SigmaPlot 12.0, and conducted phylogenetic analyses using MEGA 5.2.

## Results and Discussion

### Outputs of the calculation of thresholds

We hypothesized that the thresholds of predictions obtained using SNAP2 and PoPMuSiC 2.1 are subject to evidence-based adjustment, similar to the EVmutation threshold. The 95% sensitivity of SNAP2 was ensured by establishing a general evidence-based threshold at a level of median − 2 SD, i.e., 61 – 2 × 46.51 = −32.02. However, the use of this threshold increases the percentage of false-positive phenotype predictions from 46% to 79%, which is not acceptable. Similarly, a sensitivity of 95% for PoPMuSiC 2.1 predictions was ensured by establishing a general evidence-based threshold at a level of median − 2 SD, i.e., 1.17 – 2 × 1.08 = −1.00. However, the use of this threshold increases the percentage of false-positive phenotype predictions from 88% to 99.9%, which is not acceptable. When we combined the three prediction methods, they displayed high sensitivity but low specificity when using both the arbitrary and general evidence-based thresholds.

The absence of any agreement in the predictions of NPAVs and the existence of 58% (arbitrary thresholds) or 45% (general evidence-based thresholds) variations, which were predicted differently using the three methods, was alarming and required a more thorough adjustment of the thresholds to produce reliable prediction outcomes. Thus, we tested the application of weighted means. The application of weighted means did not exert any substantial effect on the sensitivity (92% with arbitrary thresholds or 94% with general evidence-based thresholds) but it decreased the specificity to 39% (arbitrary thresholds) and 31% (general evidence-based thresholds).

This issue would potentially be overcome by applying gene-specific evidence-based thresholds, i.e., the thresholds that were calculated individually for each analyzed gene. However, this approach did not overcome the specificity issue, as the problem associated with the incorrect detection of NPAVs remained. PoPMuSiC 2.1 was more problematic in this regard, as its predictions were so variable and skewed that the threshold set as a mean − 2 SD of DAVs often exceeded the range of predictions of NPAVs. Using this approach, PoPMuSiC 2.1 incorrectly detected 515 (24.6%) of NPAVs as associated with an effect, although the other two predictors generated correct predictions for this pool of variations. Thus, the agreement of the three methods on the non-pathogenicity of NPAVs was reached for only five (0.2%) of the 2093 NPAVs.

Next, we tested whether the implementation of two gene-specific evidence-based thresholds per predictor for each gene would be the solution. One threshold was set to 95% sensitivity (i.e., the threshold used above) and the other threshold was set to 95% specificity. When we implemented the new combination of thresholds, the three prediction methods only agreed on the predictions for the effects of 303 variations. Among these variations, 301 variations (99.3%) were DAVs and two variations (0.7%) were NPAVs. Similar to the previous approach, the problematic outcome was primarily caused by the inclusion of hypervariable predictions generated by PoPMuSiC 2.1. When we excluded PoPMuSiC 2.1 from the analyses, the gene-specific 95% specificity threshold was passed by 763 variations (11.5%), of which 752 variations (98.6%) were DAVs and 11 variations (1.4%) were NPAVs. The gene-specific 95% sensitivity threshold was passed by 622 variations (9.4%), of which 102 variations (16.4%) were DAVs and 520 variations (83.6%) were NPAVs. Thus, these findings provide proof of concept that the evidence-based adjustment of thresholds for EVmutation and SNAP2 enables the highly specific selection of both DAVs and NPAVs. To our knowledge, this approach is the first to allow the highly specific selection of variations that are not associated with any clinical phenotype. Within the tested dataset, the predictable variations accounted for 21% of the tested variations. The other variations were divided into the following three categories: a) the predictions of EVmutation and SNAP2 were contradictory (0.2%), b) one of the two predictors did not exceed either of the two thresholds (30.4%), and c) both predictors did not exceed their thresholds (48.7%). The use of weighted means combined with the two gene-specific evidence-based thresholds per predictor did not improve the outcomes and resulted in 33.5% sensitivity and 93.7% specificity.

When we analyzed the EVmutation outputs alone using the identical two gene-specific evidence-based thresholds per predictor for each gene, the gene-specific 95% specificity threshold was passed by 1236 (18.6%) variations, of which 1188 (96.1%) were DAVs and 48 (3.9%) were NPAVs. The gene-specific 95% sensitivity threshold was passed by 807 (12.2%) variations, of which 164 (20.3%) were DAVs and 643 (79.7%) were NPAVs. Thus, the use of EVmutation alone was associated with a slightly greater number of both false negative and false positive predictions, but provided a prediction for a larger percentage of the analyzed variations. Within the tested dataset, the predictable variations accounted for 31% of the total number of tested variations.

When we analyzed the SNAP2 outputs alone using the identical two gene-specific evidence-based thresholds per predictor for each gene, the gene-specific 95% specificity threshold was passed by 1390 (20.9%) of variations, of which 1343 (96.6%) were DAVs and 47 (3.4%) were NPAVs. The gene-specific 95% sensitivity threshold was passed by 1365 (20.6%) variations, of which 403 (29.5%) were DAVs and 962 (70.5%) were NPAVs. Thus, the use of SNAP2 alone was associated with a slightly greater number of both false negative and false positive predictions but provided a prediction for a larger percentage of the analyzed variations compared to its combination with EVmutation or to EVmutation alone. Within the tested dataset, the predictable variations accounted for 41% of the tested variations.

### EVmutation under default settings

The arbitrary threshold used for the EVmutation analysis enables the correct prediction of a phenotype for 99.5% of DAVs and 99.7% of partial phenotype-associated variations; this sensitivity is consistent with previously reported data^14^. However, 94.8% of NPAVs were in the same category and were predicted to exert an effect. Thus, the arbitrary zero threshold was associated with only a 5.2% specificity for clinically manifested phenotypes (Fig. 1b).

A high number of false positives was observed for all 44 analyzed genes (Fig. 1c). The EVmutation analysis provided the correct predictions of DAVs for all tested genes (median sensitivity of 100%, minimum sensitivity of 92.3% (*RET*)), but only correctly predicted a negligible fraction of NPAVs (median specificity of 4.4%, minimum specificity of 0% (12 genes), maximum specificity of 20% (*CD40LG*)).

### Tailored EVmutation thresholds

The arbitrary threshold does not provide a reliable prediction of the disease association of variations in tested genes. Therefore, we focused on whether the thresholds can be tailored either in a general or gene-specific manner. The median ± SD of predictions obtained using EVmutation for DAVs reached −6.58 ± 2.23, whereas the values for NPAVs only reached −3.86 ± 2.41. Thus, these two groups of variations were not separated to an extent that was sufficient for distinguishing between them based on, for example, their confidence intervals. Nevertheless, when focusing on the gene-specific level, the median values of predictions of the DAVs for any gene were lower than the median values of the predictions of NPAVs within the same genes. The scores and resolution varied across the analyzed genes (Fig. S1a). A sensitivity of 95% was assumed by setting the threshold to the median +2 SD of the DAVs, i.e., −6.57 + 2 × 2.22 = −2.13. Thus, the EVmutation score of −2.13 was considered a general evidence-based threshold. Its use increases the specificity to 21.5%, which is, however, still far from any reliable use of this approach.

### Constraints in VUS criteria

The VUS classification differentiates VUSs from likely benign variations based on evidence of their conservation in other mammalian species. We identified the conserved variations with the zero GV scores, i.e., variations that were conserved across the whole class of mammals, including marsupials and/or monotremes. The conserved variations represented 69.7% of NPAVs and 86.2% of DAVs. The conserved variations were associated with slightly lower EVmutation scores for both DAVs and NPAVs (Fig. 1d) compared to variations that affected evolutionarily variable sites. Nevertheless, the EVmutation scores of the four groups of variations overlapped and required further stratification. Thus, we examined the relative proportion of variations with a GV score >0 individually in each of the 44 analyzed genes (Fig. S2a). All variations in some genes displayed a zero GV score (*AR* and *PTEN*), whereas variations in other genes were poorly conserved (*ELANE*, *PROC* and *CD40LG*). Based on this finding, the arbitrary criteria for the inclusion of protein sequences in the MSAs derived from the VUS criteria were not functional since they did not reflect differences in the conservation of individual genes. Absolute values of the GV scores (degree of conservation of the respective amino acid) were not associated with any differences in clinical phenotypes (Fig. S2a) or EVmutation scores (Fig. S2b) for variations of these amino acids. However, the binary response (zero GV score *vs* any higher GV score) predicted the stratification of variations into DAVs and NPAVs.

**Figure 2:**
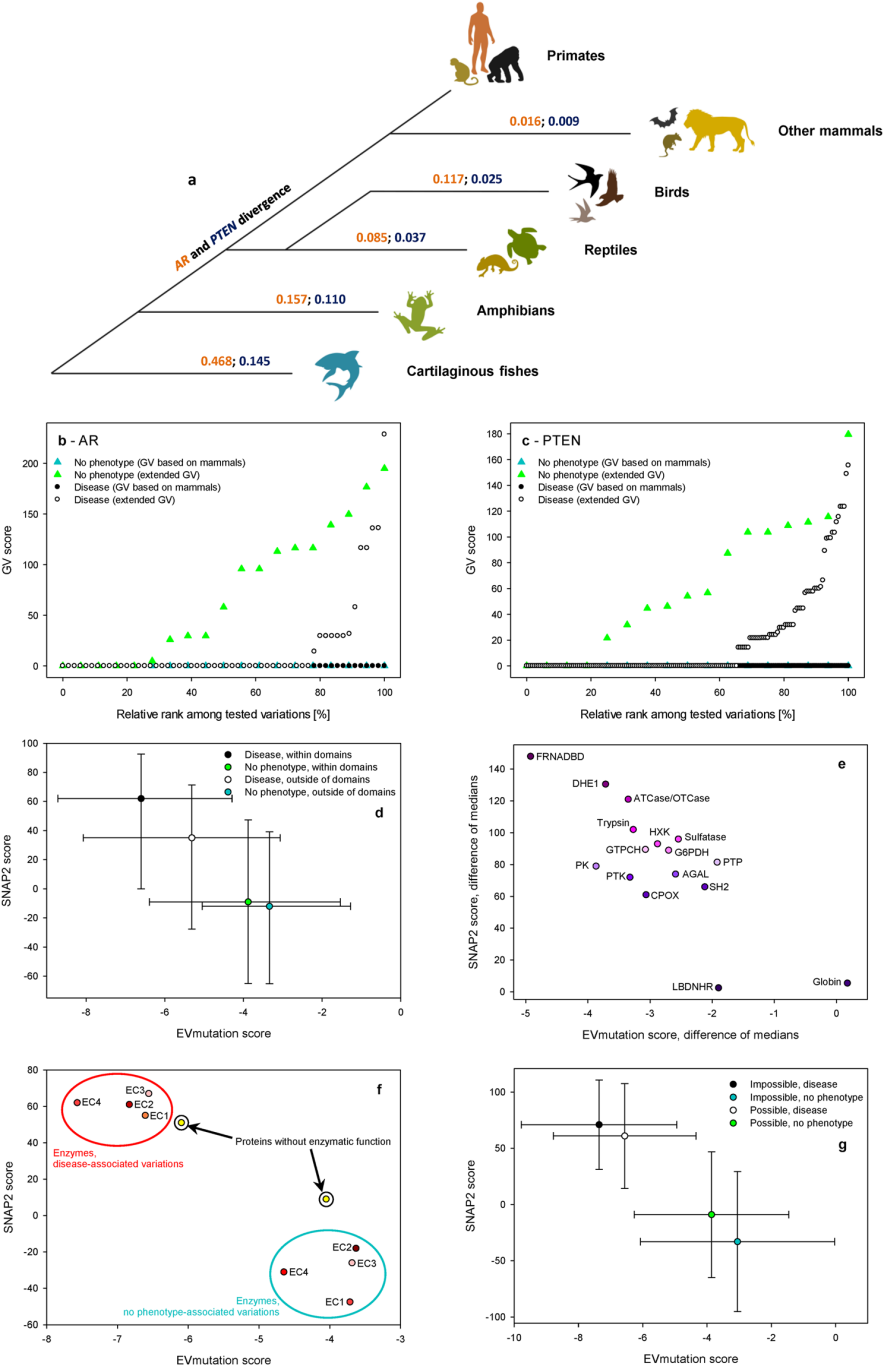
The predictions differ for evolutionarily conserved proteins, such as AR or PTEN, for variations within and outside of protein domains and for enzymes and proteins without enzymatic functions. (**a**) Evolutionary divergence of the amino acid sequences of AR and PTEN reported as the number of amino acid substitutions per site by averaging all sequence pairs between primates and other groups. (**b,c**) GV scores for amino acids within the AR (**b**) and PTEN (**c**) sequences. The data are shown separately for GV scores calculated based on mammalian protein orthologs (the two lines at the zero GV score) and extended MSAs that included more evolutionarily distant taxa. The data are shown for disease-associated and no phenotype-associated variations. Relative ranks among tested variations are shown to reflect the different numbers of variations included in each analyzed group. (**d**) EVmutation and SNAP2 scores applied to disease-associated and no phenotype-associated variations that are present or absent from protein domains. Data are presented as medians ± SD. (**e**) Differences in median EVmutation and SNAP2 scores between disease-associated and no phenotype-associated variations located within the indicated protein domains. Abbreviations for the domains: AGAL, alpha-galactosidase A; ATCase/OTCase, aspartate/ornithine carbamoyltransferase, carbamoyl-P binding and Asp/Orn binding domains; CPOX, coproporphyrinogen III oxidase; DHE1, dehydrogenase E1 component; FRNADBD, ferric reductase, NAD binding domain; GTPCH, GTP cyclohydrolase I; G6PDH, glucose-6-phosphate dehydrogenase, NAD binding and C-terminal domains; HXK, hexokinase; LBDNHR, ligand-binding domain of nuclear hormone receptor; PK, protein kinase; PTK, protein tyrosine kinase; PTP SH2, Src Homology 2 domain. (**f**) Median EVmutation and SNAP2 scores calculated for disease-associated and no phenotype-associated variations in the four indicated enzyme classes and in proteins without enzymatic functions. (**g**) EVmutation and SNAP2 scores calculated for disease-associated and no phenotype-associated variations considered possible or impossible variations according to Bromberg *et al*.^15^ Data are shown as medians ± SD.

We postulated that the MSAs, which were based on VUS inclusion criteria, were insufficient for the analyses of highly conserved genes, such as *AR* or *PTEN*, because these genes displayed low amino acid sequence divergence among their mammalian orthologs. The solutions consisted of the addition of more evolutionarily distant taxa into the alignments (Figs. 2a and S3). This addition increases the divergence between the analyzed groups of organisms (Tables S1, S2), which is sufficient to generate a pool of informative amino acids that are susceptible to variations during the course of evolution. Although the VUS-based GV score (i.e., the score that was based solely on sequences of mammalian orthologs) did not discriminate between the DAVs and NPAVs, the GV score based on extended MSAs led to a clear differentiation between DAVs and NPAVs. The DAVs were associated with 60–80% of amino acids with a zero GV score. In contrast, the NPAVs reached zero scores in 20–30% of cases (Fig. 2b,c). Thus, the MSAs used to calculate the GV scores of highly conserved proteins were improved by including sequences from evolutionarily distant organisms until an experimentally or arbitrarily set value of sequence divergence between analyzed groups (≥0.1 substitutions per amino acid) was achieved. Even using these improved settings, a large group of variations were considered DAVs, despite displaying high GV scores (Fig. 2b,c).

### Combination of EVmutation with methods based on different approaches

We next focused on improving EVmutation-based predictions by combining them with other state-of-the-art prediction methods that provide numerical outcomes and thresholds, which can easily undergo evidence-based adjustment. Similar to EVmutation, the arbitrary settings of SNAP2^20^ and PoPMuSiC 2.1^13^ do not correspond to the division of clinically observed variations into DAVs and NPAVs (Fig. S4a,b). For SNAP2, 84% of predictions of DAVs and 54% of predictions of NPAVs were correct. Thus, the percentage of true disease predictions was slightly lower than with EVmutation, but the percentage of true no phenotype predictions was higher by an order of magnitude than with EVmutation. For PoPMuSiC 2.1, we obtained correct predictions for 94% of DAVs and only 12% of NPAVs. Thus, the number of true disease predictions was slightly lower than with EVmutation, and the percentage of true no phenotype predictions was similar to EVmutation. In contrast to EVmutation, the latter two prediction methods were associated with a high variability of predictions between the analyzed proteins (Fig. S4c,d).

We hypothesized that the thresholds of predictions obtained using SNAP2 and PoPMuSiC 2.1 could benefit from being subjected to evidence-based adjustment, similar to the adjustment of the EVmutation threshold. We tested several approaches for calculating the thresholds (see the chapter Outputs of the calculation of thresholds for a detailed description of the applied approaches), but most of these approaches only provided minor or no improvements. Additionally, the PoPMuSiC 2.1 scores were associated with such high overlap of the distribution of DAVs and NPAVs that the outcomes of this method were uninformative. Therefore, we excluded PoPMuSiC 2.1 from further analyses. The approach that led to a substantial improvement in the credibility of predictions was the implementation of two gene-specific evidence-based thresholds per predictor for each gene. One gene-specific threshold was set to 95% sensitivity (i.e., the threshold used above) and the other threshold was set to 95% specificity. For the combination of EVmutation and SNAP2, the predictable variations represented 21% of the total number of tested variations. The predictions were associated with 98.6% specificity and 83.6% sensitivity. Thus, this result serves as proof of concept that the evidence-based adjustment of thresholds for EVmutation and SNAP2 enables the highly specific selection of both DAVs and NPAVs. To our knowledge, this approach is the first to enable the highly specific selection of variations that are not associated with any clinical phenotype.

When the two predictors were used alone, the percentage of predictable variations increased (to 31% using EVmutation and 41% using SNAP2), but the specificity and sensitivity decreased. For EVmutation, the specificity was 96.1% and sensitivity was 79.7%. For SNAP2, the specificity was 96.6% and sensitivity was 70.5%. Thus, the use of EVmutation or SNAP2 alone was associated with a slightly higher number of both false negative and false positive predictions but provided a prediction for a larger percentage of the analyzed variations when compared to their combination.

### Factors contributing to the variability within the analyzed dataset

The predictions of the effects of DAVs and NPAVs differed for variations located within or outside of the protein domains (*t*-test *p* < 0.001 each, for EVmutation and SNAP2, respectively). The predictions of the effects of DAVs differed for variations located within and outside of the protein domains (*t*-test *p* < 0.001 each, for EVmutation and SNAP2, respectively). In contrast, the NPAVs did not display any significant difference between their pools located within and outside of the protein domains (*t*-test *p* > 0.05 each, for EVmutation and SNAP2, respectively) (Fig. 2d). Thus, the predictions of the variations present within protein domains displayed a higher amplitude (EVmutation −2.722 *vs* −1.973, and SNAP2 71 *vs* 47). When focusing on particular domain types, the differences between DAVs and NPAVs were significant for all major domain types (*t*-test with the Bonferroni’s correction *p* < 0.001), except the globin domain (*t*-test with the Bonferroni’s correction *p* > 0.05 for both predictors) and ligand-binding domain of nuclear hormone receptor (SNAP2 *t*-test with the Bonferroni’s correction *p* > 0.05) (Fig. 2e and Table S3). In the combination approach, the variations that were located within catalytically active protein domains (e.g., tyrosine kinases or serine-threonine kinases) were easier to predict than variations that were located outside of any domains. The prediction of variations located within certain protein domains lacking intrinsic enzymatic activity was highly problematic, but certain enzymatically inactive domains (e.g., the SH2 domain) were still associated with an acceptable resolution of the predictions. The rigidity of the SH2 domain structure (needed for pTyr binding)^36^ was likely responsible for this difference in prediction outcomes compared with the globin domains. The globin domains maintain their function, regardless of their low sequence identity, as long as the hydrophobic core and hydrophilic surface are maintained^37^. The predictions of variations in the amino acid sequences of enzymes also showed a better resolution than those of variations located in proteins without enzymatic functions (Fig. 2f). Only differences between the DAVs (but not NPAVs) of proteins without enzymatic function and any of the four enzyme classes tested were significant (Kruskal-Wallis one-way ANOVA on ranks with Dunn’s post-tests *p* < 0.05 each; Table S4). Future algorithms should match the predictions with protein attributes, such as the presence of specific protein domains^38^. The binary presence/absence information for the location in protein domains is used to identify driver and passenger somatic mutations involved in oncogenesis^39^ and has been reflected in several prediction systems^40^. Methods designed to account for the specific characteristics of particular domain types should be considered an integral part of prediction algorithms (Fig. 2e).

According to previous studies, that amino acid variations that are caused by single nucleotide polymorphisms (“possible” variations) are slightly less deleterious than variations that occur when two or three nucleotides within the affected triplet are substituted (“impossible” variations)^15^. Although the likelihood of impossible variations occurring was low, we identified 97 (1.5%) of these variations within the analyzed dataset. Among impossible variations, we did not observe a significant improvement in the resolution of DAVs and NPAVs (Kruskal-Wallis one-way ANOVA on ranks, with Dunn’s post-tests, *p* > 0.05 each). The DAVs were equally frequent among impossible (71%) and possible (68%) variations (χ^2^ test *p* > 0.05 when the data were normalized to the total number of impossible variations) (Fig. 2g).

**Figure 3:**
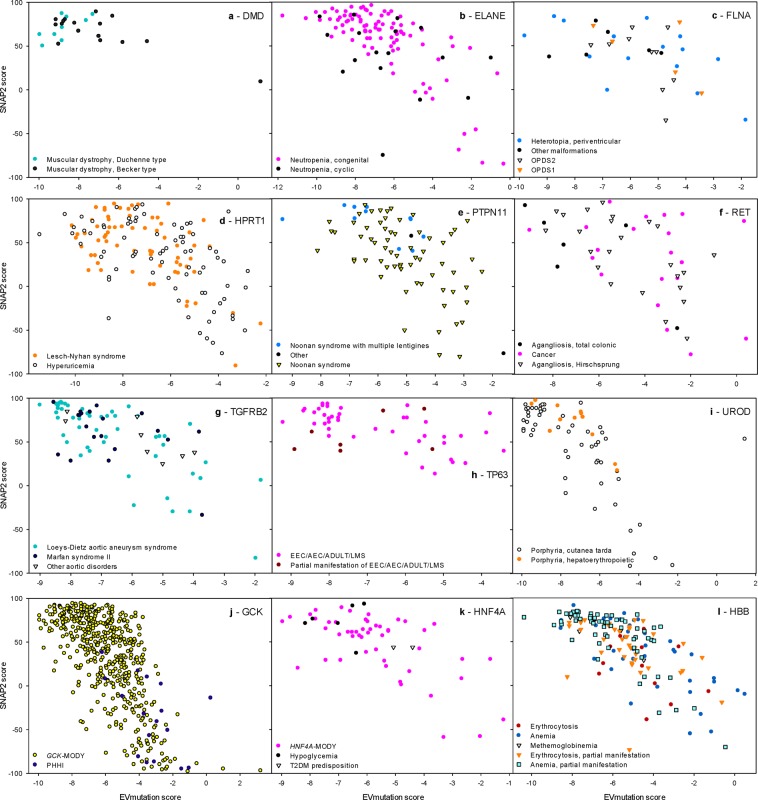
The efficiency of the prediction methods in discriminating among multiple diseases caused by missense variations in the indicated proteins. EVmutation and SNAP2 scores are shown for proteins with significantly different disease-specific scores (**a–i**) or that result in the opposite phenotypes (**j–l**). (**a**) *DMD*, (**b**) *ELANE*, (**c**) *FLNA*, (**d**) *HPRT1*, (**e**) *PTPN11*, (**f**) *RET*, (**g**) *TGFRB2*, (**h**) *TP63*, (**i**) *UROD*, (**j**) *GCK*, (**k**) *HNF4A*, and (**l**) *HBB*.

Because the effects of DAVs were not predicted by arbitrary thresholds, but by gene-specific thresholds (Figs. 1 and 3), we hypothesized that the prediction methods would differentiate between multiple diseases caused by variations in a single protein. Dunn’s and Tukey’s post-tests indicated the possibility of such differential diagnoses in several proteins (see Table S5 for an overview of outputs of statistical tests). We plotted the EVmutation and SNAP2 prediction scores for DAVs in nine proteins, for which the variations associated with the multiple phenotypes statistically differ (Fig. 3a–i), and for two proteins (*GCK* and *HNF4A*) in which variations cause opposite phenotypes, i.e., diabetes and hyperglycemia (Fig. 3j,k) or (*HBB*) erythrocytosis and anemia (Fig. 3l). Despite the statistically significant differences, the variability in predictions of the genes prevented the assignment of the variations to particular diseases, except for extreme values. Examples are listed below: (a) The EVmutation score of *DMD* > −7 predicts muscular dystrophy of the Becker type (Fig. 3a). (b) Noonan syndrome with multiple lentigines is associated with variations with an EVmutation score for *PTPN11* < −4 and a SNAP2 score for *PTPN11* > 30 (Fig. 3e). (c) The EV mutation score for *UROD* > −4 or the SNAP2 score for *UROD* < 0 predict the manifestation of porphyria cutanea tarda instead of hepatoerythropoietic porphyria (Fig. 3i).

### Identification of variations under negative selection

We then used the newly obtained evidence-based knowledge to predict theoretically possible variations that have never been encountered in the clinic. This approach might highlight critical constrained variations that have not yet been linked to human disease phenotypes. Some of these variations likely exhibit such extreme constraints because they lead to extreme developmental disorders, are embryonically lethal or cause a long-term selection pressure by decreasing the fitness of their carriers. Although the theoretical ratio of impossible to possible variations was 2.47:1, the clinically observed ratio was 0.0143:1. The impossible and possible variations differed significantly in the scores obtained from both predictors (*t*-test *p < *0.001 each), with EVmutation scores reaching −6.00 ± 2.42 and −4.83 ± 2.49, and SNAP2 scores reaching 40 ± 51 and 18 ± 56 for impossible and possible variations, respectively. The gene-specific comparisons of the distribution of scores of impossible and possible variations and their comparison with the distribution of clinically documented DAVs and NPAVs are provided in Fig. S5.

The previous single-gene-oriented case study identified the potential existence of a pool of underrepresented variations in both healthy and disease-affected variation carriers^8^. Since the present study provides the first large-scale adjustment of prediction scores based on clinical data, we focused on the detection of variations undergoing negative selection during molecular evolution. When performing this analysis (and in contrast to the aforementioned case study)^8^, we excluded any variations considered impossible by Bromberg *et al*.^15^ and analyzed the similarities of distributions of DAVs and possible theoretical variations. For simplicity, we compared the positions of the 10^th^ percentiles for EVmutation scores and 90^th^ percentiles for SNAP2 scores, which represent the predictions of amino acid changes with the most deleterious effects on proteins. Since possible theoretical variations include both putative DAVs and NPAVs, we expected that the analyzed values calculated based on possible theoretical variations should be closer towards the scores of NPAVs. The differences in the 10^th^ percentiles of EVmutation scores ranged from −1.093 to 3.360 (mean 0.921) and the differences in the 90^th^ percentiles of SNAP2 scores ranged from −25.0 to 2.6 (mean −11.5).

**Figure 4:**
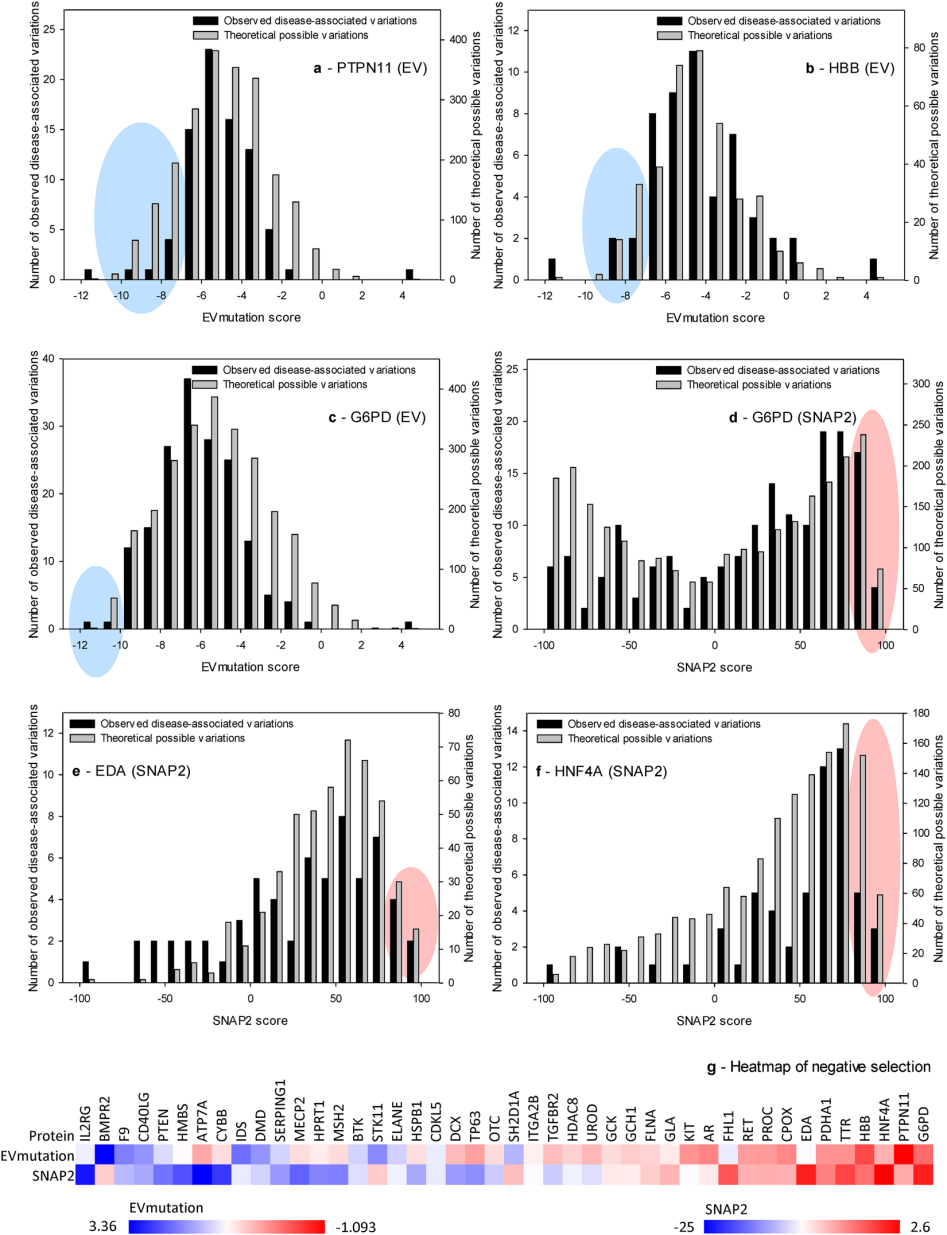
The detection of variations under negative selection during molecular evolution: an example of the application of evidence-based knowledge. (**a–f**) The distribution of observed disease-associated variations compared to the distribution of possible^10^ theoretical variations. The data are shown for the proteins for which negative values were obtained from the calculation of the differences in the 10^th^ percentiles of EVmutation scores – (**a**) *PTPN11*, (**b**) *HBB* and (**c**) *G6PD* – and for genes for which positive values were obtained from the calculation of the differences in 90^th^ percentiles of SNAP2 scores – (**d**) *G6PD*, (**e**) *HNF4A* and (**f**) *EDA*. (**g**) The heatmap of proteins causing Mendelian diseases sorted according to the likelihood that their variations included variations that were under negative selection during molecular evolution. Ranges of differences in median values: from −1.093 to 3.36 (EVmutation) and from −25 to 2.6 (SNAP2).

In three genes (*PTPN11*, *HBB* and *G6PD*), the positions of 10^th^ percentiles of the EVmutation scores were lower for DAVs than possible theoretical variations in the same genes. Similarly, in three genes (again *G6PD*, but also *HNF4A* and *EDA*) the positions of 90^th^ percentiles of the SNAP2 scores were higher for DAVs than possible theoretical variations in the same genes. Thus, the variations that were predicted to be the most deleterious by EVmutation and/or SNAP2 were substantially depleted among DAVs compared to the spectra of possible theoretical variations in the same genes. These variations were therefore underrepresented among disease-affected variation carriers (Fig. 4a–f) and were under negative selection during molecular evolution. The heatmap of analyzed proteins, which were sorted according to the likelihood that their variations included variations under negative selection during molecular evolution, is shown in Fig. 4g. The phenotypes that are commonly associated with variations in these five genes are listed in Table 2. Confirmation of the negative selection against the underrepresented variations should consist of a series of studies that would compare the *in vitro* or *in vivo* effects of theoretical variations, which were hypothesized to be under negative selection, with clinically observed variations, which were within the range that did not seem to be subject to negative selection. During the peer-review of this manuscript, Havrilla *et al*.^41^ published a detailed map of constrained coding regions (CCR) in human genes and revealed that the most constrained regions are located in known disease loci. The genes encoding proteins associated with Mendelian diseases that we identified by applying the 10^th^/90^th^ percentiles of DAVs partially overlapped with genes that ranked highly in the study by Havrilla *et al*.^41^. Namely, the CCR percentiles were 95.2% – 97.8% for *PTPN11* and 97.8% for *HNF4A*. However, other genes, namely *HBB*, *G6PD* and *EDA* were not among top hits in the previous CCR study.

**Table 2:** Major phenotypes associated with genes that were underrepresented among disease-affected carriers.

Gene	Phenotype	References
*PTPN11*	Multiple lentigines / LEOPARD syndrome	^55–61^
	Noonan syndrome	^62–64^
*HBB*	Thalassemia beta	^65–67^
	Hemolytic anemia	^68–70^
	Erythrocytosis	^71–73^
*G6PD*	Glucose-6-phosphate dehydrogenase deficiency	^74–76^
*HNF4A*	Hypoglycemia, hyperinsulinemic	^77–79^
	Diabetes, *HNF4A*-MODY	^79–81^
*EDA*	Oligodontia	^82–84^
	Ectodermal dysplasia, hypohidrotic	^85–87^
	Ectodermal dysplasia	^88–90^

See Table S7 for a complete list of phenotypes associated with analyzed variations and source references.

### Validation and conclusions

**Figure 5:**
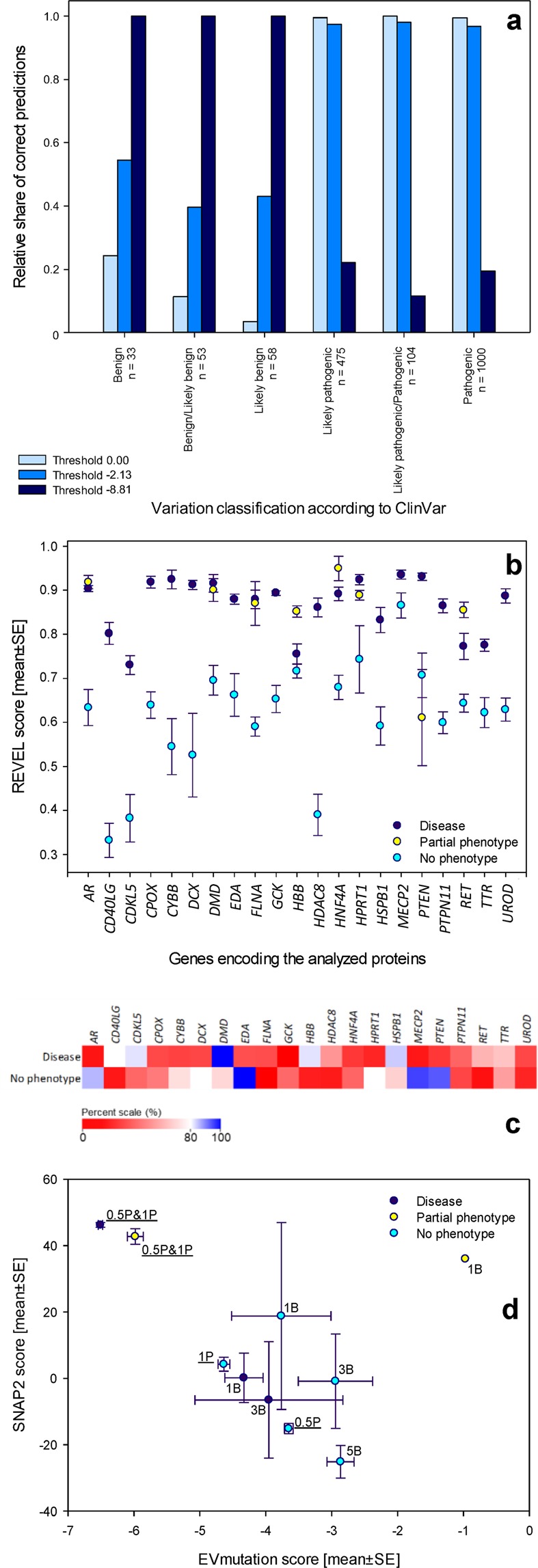
Validation of the model, identification of the specificity of the consensus classifier REVEL, and the application of the American College of Medical Genetics and Genomics (ACMG) criteria for the classification of variations. (**a**) Validation of the threshold values for EVmutation that were suggested in the proposed model. Validation was performed using a set of 1723 variations in 63 genes (Tables S8–S10), which were classified according to ClinVar. The data are presented as relative percentages of correct predictions using the arbitrary EVmutation threshold (0.00), the evidence-based threshold that allows 95% sensitivity (−2.13) and the threshold that allows 95% specificity (−8.81). (**b,c**) REVEL, a consensus classifier, is associated with the issue of low specificity, similar to the individual computational algorithms. REVEL scores were retrieved for a set of 2721 variations in 21 genes. Mean REVEL scores for the individual genes discriminated well between the disease-associated and no phenotype-associated variations (**b**). However, because a large overlap in the predictions was observed, the specificity was low for most of the analyzed genes (**c**). Data are presented (**b**) as the means ± SE or (**c**) as relative percentages of correct predictions of the association of the variations with diseases (upper row) or no phenotypes (lower row). (**d**) Application of the ACMG criteria for the classification of variations, which classify the variations as benign (1B and higher) and pathogenic (0.5 P and higher) according to the population frequencies of the variations (Table S11). The EVmutation and SNAP2 scores were analyzed separately for the disease- and no phenotype-associated variations. Data are shown as means ± SE.

We validated the threshold values for EVmutation scores that were suggested in the proposed model. We established an independent dataset of variations in genes associated with Mendelian diseases (Tables S8, S9). The tested variations were classified according to ClinVar. The mean EVmutation scores for pathogenic and benign variations were consistently below their previously suggested zero threshold (Table S10). The shift of the general EVmutation threshold to −2.13 led to a similar and significant improvement in the specificity of predictions of benign and likely benign variations, while the sensitivity remained higher than 96% for the pathogenic variations (Fig. 5a).

We calculated the sensitivity and specificity of the predictions retrieved from REVEL to determine whether the issue of low specificity was specifically associated with the outcomes of individual computational algorithms, such as EVmutation, or whether it also affected the data obtained from state-of-the-art consensus classifiers^5^. REVEL predictions exhibited similar issues to the individual predictors. The scores for DAVs and NPAVs were gene-specific (Fig. 5b). The specificity was both low and gene-specific (Fig. 5c). Thus, although despite the consensus classifiers have the potential to eliminate the errors generated by individual predictors, they were prone to the systemic issue of low specificity.

All studies of human variations have a limitation in terms of how the variations are classified. For example, the incomplete penetrance may cause errors in the classification of rare variations^42^. We re-analyzed the EVmutation and SNAP2 scores based on the ACMG criteria for the classification of variations to corroborate the key outcomes of the present study (Fig. 5d)^24^. Variations classified as pathogenic according to the ACMG criteria were identified in both the DAV and NPAV datasets. EVmutation and SNAP2 identified only the first of these two groups as pathogenic. This difference in predictions was absent for common and rare variations among the NPAVs, which may reflect possible biases in the training or testing datasets for both of these methods^14,20^.

The outcomes of prediction methods are often uncritically used, particularly by non-specialists in the field, who benefit from their use for the purpose of narrowing the number of hits identified during omics screens performed for scientific or clinical purposes. The uncritical use of the prediction methods is facilitated by including them in the tools commonly used for these purposes, such as the inclusion of SIFT and PolyPhen algorithms in the Ensembl genome browser (http://www.ensembl.org/; Release 90 cited). Based on accumulating evidence, the prediction methods are often over-interpreted, mainly because they exhibit high false positive rates^8,43^, and sufficiently complex datasets used for the design, testing and training of the methods are lacking^44^. Any distinct effects observed at the molecular level depend on the context and can be compensated by intrinsic regulatory pathways of the organism, which particularly applies to the effects of variations in nonessential peripheral enzymes and signaling proteins^14,45,46^.

New prediction methods are rapidly released, and EVmutation is one of the most recent contributions to the field^14^. EVmutation is important because it includes epistasis when modeling the effect of the respective variation. We provided the first match for the EVmutation (and SNAP2 and PoPMuSiC 2.1) prediction outcomes with clinical phenotypes of a large pool of pathogenic and benign variations in genes associated with Mendelian diseases. EVmutation, similar to the other tested prediction methods, had high sensitivity but also extremely low specificity. We suggested the use of evidence-based thresholds, which were obtained by calculating and testing several variants of the thresholds until we reached 98.6% sensitivity and 83.6% specificity, leaving the certain pool of variations unresolved (if needed, the size of this pool can be decreased at the cost of decreasing the sensitivity and/or specificity). The predictions provided better resolution for variations located in enzymes and predominantly those within enzymatic domains. For some proteins, the use of numerical outputs of predictions combined with evidence-based thresholds distinguished between multiple diseases caused by variations in the same protein. We identified large previously unreported pools of variations that underwent negative selection during molecular evolution and were absent in patients. These variations were particularly prominent in *G6PD*, *PTPN11*, *HNF4A* and *HBB*. Further research should focus on the use of evidence-based thresholds for categories of variations defined using the Human Phenotype Ontology (such as the Phenomizer or Phevor)^47,48^ and phenome-wide association studies (PheWAS)^49,50^.

Based on the large-scale analysis provided in the present study, we suggest the use of evidence-based thresholds to improve the outcomes of any prediction methods that produce numerical scores. Improved settings of the individual methods will facilitate the outcomes of consensus classifiers represented by REVEL^5^, PredictSNP^51^, PredictSNP2^52^, CADD^53^ or DANN^54^. The evolutionary variation analysis approach described here is the first to enable the highly specific identification of likely disease-causing missense variations that have not yet been associated with any clinical phenotype.

## Supplementary information

Supplementary Appendix 1

Supplementary Appendix 2

## Data Availability

All data are available in the main text or in the Supplementary Materials.

## References

[1] Biesecker LG, Green RC (2014). Diagnostic clinical genome and exome sequencing. N. Engl. J. Med..

[2] Simm F (2018). Identification of SLC20A1 and SLC15A4 among other genes as potential risk factors for combined pituitary hormone deficiency. Genet. Med..

[3] Tennessen JA (2012). Evolution and functional impact of rare coding variation from deep sequencing of human exomes. Science.

[4] The 1000 Genomes Project Consortium (2012). An integrated map of genetic variation from 1,092 human genomes. Nature.

[5] Ioannidis NM (2016). REVEL: an ensemble method for predicting the pathogenicity of rare missense variants. Am. J. Hum. Genet..

[6] Cirulli ET, Goldstein DB (2010). Uncovering the roles of rare variants in common disease through whole-genome sequencing. Nat. Rev. Genet..

[7] Bamshad MJ (2011). Exome sequencing as a tool for Mendelian disease gene discovery. Nat. Rev. Genet..

[8] Šimčíková D, Kocková L, Vackářová K, Těšínský M, Heneberg P (2017). Evidence-based tailoring of bioinformatics approaches to optimize methods that predict the effects of nonsynonymous amino acid substitutions in glucokinase. Sci. Rep..

[9] Hayat S, Sander C, Marks DS, Elofsson A (2015). All-atom 3D structure prediction of transmembrane β-barrel proteins from sequences. Proc. Natl. Acad. Sci. USA.

[10] Wang Y, Barth P (2015). Evolutionary-guided de novo structure prediction of self-associated transmembrane helical proteins with near-atomic accuracy. Nat. Commun..

[11] Peled S (2016). *De-novo* protein function prediction using DNA binding and RNA binding proteins as a test case. Nat. Commun..

[12] Huang YF, Gulko B, Siepel A (2017). Fast, scalable prediction of deleterious noncoding variants from functional and population genomic data. Nat. Genet..

[13] Dehouck Y, Kwasigroch JM, Gilis D, Rooman M (2011). PoPMuSiC 2.1: a web server for the estimation of protein stability changes upon mutation and sequence optimality. BMC Bioinform..

[14] Hopf TA (2017). Mutation effects predicted from sequence co-variation. Nat. Biotechnol..

[15] Bromberg Y, Kahn PC, Rost B (2013). Neutral and weakly nonneutral sequence variants may define individuality. Proc. Natl. Acad. Sci. USA.

[16] Vaser R, Adusumalli S, Leng SN, Sikic M, Ng PC (2016). SIFT missense predictions for genomes. Nat. Protoc..

[17] Libbrecht MW (2015). Machine learning in genetics and genomics. Nat. Rev. Genet..

[18] Sela I, Ashkenazy H, Katoh K, Pupko T (2015). GUIDANCE2: accurate detection of unreliable alignment regions accounting for the uncertainty of multiple parameters. Nucl. Acids Res..

[19] Adebali O, Reznik AO, Ory DS, Zhulin IB (2016). Establishing the precise evolutionary history of a gene improves prediction of disease-causing missense mutations. Genet. Med..

[20] Hecht M, Bromberg Y, Rost B (2015). Better prediction of functional effects for sequence variants. BMC Genom..

[21] DePristo MA, Weinreich DM, Hartl DL (2005). Missense meanderings in sequence space: a biophysical view of protein evolution. Nat. Rev. Genet..

[22] de Visser JA, Krug J (2014). Empirical fitness landscapes and the predictability of evolution. Nat. Rev. Genet..

[23] Breen MS, Kemena C, Vlasov PK, Notredame C, Kondrashov FA (2012). Epistasis as the primary factor in molecular evolution. Nature.

[24] Nykamp K (2017). Sherloc: a comprehensive refinement of the ACMG-AMP variant classification criteria. Genet. Med..

[25] Richards S (2015). Standards and guidelines for the interpretation of sequence variants: a joint consensus recommendation of the American College of Medical Genetics and Genomics and the Association for Molecular Pathology. Genet. Med..

[26] Stenson PD (2014). The Human Gene Mutation Database: building a comprehensive mutation repository for clinical and molecular genetics, diagnostic testing and personalized genomic medicine. Hum. Genet..

[27] Liu L (2009). High-density SNP genotyping to define beta-globin locus haplotypes. Blood Cells Mol. Dis..

[28] Steele AM (2011). The previously reported T342P GCK missense variant is not a pathogenic mutation causing MODY. Diabetologia.

[29] Chellapa K (2012). Src tyrosine kinase phosphorylation of nuclear receptor HNF4α correlates with isoform-specific loss of HNF4α in human colon cancer. Proc. Natl. Acad. Sci. USA.

[30] Houlleberghs H (2016). Oligonucleotide-directed mutagenesis screen to identify pathogenic Lynch syndrome-associated MSH2 DNA mismatch repair gene variants. Proc. Natl. Acad. Sci. USA.

[31] Maxwell KN (2016). Evaluation of ACMG-guideline based variant classification of cancer susceptibility and non-cancer-associated genes in families affected by breast cancer. Am. J. Hum. Genet..

[32] Walsh R (2016). Reassessment of Mendelian gene pathogenicity using 7,855 cardiomyopathy cases and 60,706 reference samples. Genet. Med..

[33] Hicks S, Wheeler DA, Plon SE, Kimmel M (2011). Prediction of missense mutation functionality depends on both the algorithm and sequence alignment employed. Hum. Mutat..

[34] Riera C, Padilla N, de la Cruz X (2016). The complementarity between protein-specific and general pathogenicity predictors for amino acid substitutions. Hum. Mutat..

[35] Mathe E (2006). Computational approaches for predicting the biological effect of p53 missense mutations: a comparison of three sequence analysis based methods. Nucl. Acids Res..

[36] Pawson T (1995). Protein modules and signaling networks. Nature.

[37] Aronson HE, Royer WE, Hendrickson WA (1994). Quantification of tertiary structural conservation despite primary sequence drift in the globin fold. Protein Sci..

[38] Rost B (2002). Enzyme function less conserved than anticipated. J. Mol. Biol..

[39] Miller ML (2015). Pan-cancer analysis of mutation hotspots in protein domains. Cell Systems.

[40] Salgado D (2016). UMD-Predictor: a high-throughput sequencing compliant system for pathogenicity prediction of any human cDNA substitution. Hum. Mutat..

[41] Havrilla JM, Pedersen BS, Layer RM, Quinlan AR (2019). A map of constrained coding regions in the human genome. Nat. Genet..

[42] Bastarache L (2018). Phenotype risk scores identify patients with unrecognized Mendelian disease patterns. Science.

[43] Romeo S (2009). Rare loss-of-function mutations in ANGPTL family members contribute to plasma triglyceride levels in humans. J. Clin. Invest..

[44] Rost B, Radivojac P, Bromberg Y (2016). Protein function in precision medicine: deep understanding with machine learning. FEBS Lett..

[45] Fowler DM, Fields S (2014). Deep mutational scanning: a new style of protein science. Nat. Methods.

[46] Boucher JI, Bolon DN, Tawfik DS (2016). Quantifying and understanding the fitness effects of protein mutations: Laboratory versus nature. Protein Sci..

[47] Singleton MV (2014). Phevor combines multiple biomedical ontologies for accurate identification of disease-causing alleles in single individuals and small nuclear families. Am. J. Hum. Genet..

[48] Bone WP (2016). Computational evaluation of exome sequence data using human and model organism phenotypes improves diagnostic efficiency. Genet. Med..

[49] Simonti CN (2016). The phenotypic legacy of admixture between modern humans and Neadertals. Science.

[50] Posey JE (2017). Resolution of disease phenotypes resulting from multilocus genomic variation. N. Engl. J. Med..

[51] Bendl J (2014). PredictSNP: robust and accurate consensus classifier for prediction of disease-related mutations. PLoS Comput. Biol..

[52] Bendl J (2016). PredictSNP2: a unified platform for accurately evaluating SNP effects by exploiting the different characteristics of variants in distinct genomic regions. PLoS Comput. Biol..

[53] Kircher M (2014). A general framework for estimating the relative pathogenicity of human genetic variants. Nat. Genet..

[54] Quang D, Chen Y, Xie X (2015). DANN: a deep learning approach for annotating the pathogenicity of genetic variants. Bioinformatics.

[55] Sarkozy A (2004). Clinical and molecular analysis of 30 patients with multiple lentigines LEOPARD syndrome. J. Med. Genet..

[56] Yoshida R (2004). Two novel and one recurrent *PTPN11* mutations in LEOPARD syndrome. Am. J. Med. Genet. A.

[57] Osawa R (2009). A novel PTPN11 missense mutation in a patient with LEOPARD syndrome. Br. J. Dermatol..

[58] Digilio MC (2002). Grouping of multiple-lentigines/LEOPARD and Noonan syndromes on the PTPN11 gene. Am. J. Hum. Genet..

[59] Seishima M (2007). Malignant melanoma in a woman with LEOPARD syndrome: identification of a germline *PTPN11* mutation and a somatic *BRAF* mutation. Br. J. Dermatol..

[60] Conti E (2003). A novel PTPN11 mutation in LEOPARD syndrome. Hum. Mutat..

[61] Keren B (2004). *PTPN11* mutations in patients with LEOPARD syndrome: a French multicentric experience. J. Med. Genet..

[62] Sarkozy A (2003). Correlation between *PTPN11* gene mutations and congenital heart defects in Noonan and LEOPARD syndromes. J. Med. Genet..

[63] Atik T (2016). Mutation spectrum and phenotypic features in Noonan syndrome with *PTPN11* mutations: definition of two novel mutations. Indian J. Pediatr..

[64] Tartaglia M (2002). *PTPN11* mutations in Noonan syndrome: molecular spectrum, genotype-phenotype correlation, and phenotypic heterogeneity. Am. J. Hum. Genet..

[65] Al-Gazali L, Ali BR (2010). Mutations of a country: a mutation review of single gene disorders in the United Arab Emirates (UAE). Hum. Mutat..

[66] Knott M (2006). Novel and Mediterranean beta thalassemia mutations in the indigenous Northern Ireland population. Blood Cells Mol. Dis..

[67] Colah R (2009). Regional heterogeneity of beta-thalassemia mutations in the multi ethnic Indian population. Blood Cells Mol. Dis..

[68] Villegas A (2003). Hb Santander [beta34(B16)Val–> Asp (GTC–> GAC)]: a new unstable variant found as a de novo mutation in a Spanish patient. Hemoglobin.

[69] Henderson SJ (2016). Ten years of routine α- and β-globin gene sequencing in UK hemoglobinopathy referrals reveals 60 novel mutations. Hemoglobin.

[70] Zanella-Cleon I (2009). Strategy for identification by mass spectrometry of a new human hemoglobin variant with two mutations in Cis in the beta-globin chain: Hb S-Clichy [beta6(A3)Glu–>Val; beta8(A5)Lys–>Thr]. Hemoglobin.

[71] Wajcman H (2003). Two new hemoglobin variants with increased oxygen affinity: Hb Nantes [beta34(B16)Val–>Leu] and Hb Vexin [beta116(G18)His–>Leu]. Hemoglobin.

[72] McClure RF, Hoyer JD, Mai M (2006). The JAK2 V617F mutation is absent in patients with erythrocytosis due to high oxygen affinity hemoglobin variants. Hemoglobin.

[73] Shin SY, Bang SM, Kim HJ (2016). A novel hemoglobin variant associated with congenital erythrocytosis: Hb Seoul [β86(F2)Ala→Thr] (HBB:c.259G>A). Ann. Clin. Lab. Sci..

[74] Vulliamy T, Beutler E, Luzzatto L (1993). Variants of glucose-6-phosphate dehydrogenase are due to missense mutations spread throughout the coding region of the gene. Hum. Mutat..

[75] Bulliamy T, Luzzatto L, Hirono A, Beutler E (1997). Hematologically important mutations: glucose-6-phosphate dehydrogenase. Blood Cells Mol. Dis..

[76] Yan T (2006). Incidence and complete molecular characterization of glucose-6-phosphate dehydrogenase deficiency in the Guangxi Zhuang autonomous region of southern China: description of four novel mutations. Haematologica.

[77] McGlacken-Byrne SM (2014). The evolving course of *HNF4A* hyperinsulinaemic hypoglycaemia–a case series. Diabet. Med..

[78] Flanagan SE (2010). Diazoxide-responsive hyperinsulinemic hypoglycemia caused by *HNF4A* gene mutations. Eur. J. Endocrinol..

[79] Colclough K, Bellanne-Chantelot C, Saint-Martin C, Flanagan SE, Ellard S (2013). Mutations in the genes encoding the transcription factors hepatocyte nuclear factor 1 alpha and 4 alpha in maturity-onset diabetes of the young and hyperinsulinemic hypoglycemia. Hum. Mutat..

[80] Urbanová J (2014). Positivity for islet cell autoantibodies in patients with monogenic diabetes is associated with later diabetes onset and higher HbA1c level. Diabet. Med..

[81] Harries LW (2008). The diabetic phenotype in *HNF4A* mutation carriers is moderated by the expression of *HNF4A* isoforms from the P1 promoter during fetal development. Diabetes.

[82] Song S (2009). *EDA* gene mutations underlie non-syndromic oligodontia. J. Dent. Res..

[83] Lee KE (2014). Oligodontia and curly hair occur with ectodysplasin-a mutations. J. Dent. Res..

[84] Ruiz-Heiland G (2016). Novel missense mutation in the *EDA* gene in a family affected by oligodontia. J. Orofac. Orthop..

[85] Cluzeau C (2011). Only four genes (*EDA1*, *EDAR*, *EDARADD*, and *WNT10A*) account for 90% of hypohidrotic/anhidrotic ectodermal dysplasia cases. Hum. Mutat..

[86] Guazzarotti L (2015). Phenotypic heterogeneity and mutational spectrum in a cohort of 45 Italian males subjects with X-linked ectodermal dysplasia. Clin. Genet..

[87] Clauss F (2010). X-linked and autosomal recessive Hypohidrotic Ectodermal Dysplasia: genotypic-dental phenotypic findings. Clin. Genet..

[88] Monreal AW, Zonana J, Ferguson B (1998). Identification of a new splice form of the *EDA1* gene permits detection of nearly all X-linked hypohidrotic ectodermal dysplasia mutations. Am. J. Hum. Genet..

[89] Schneider P (2001). Mutations leading to X-linked hypohidrotic ectodermal dysplasia affect three major functional domains in the tumor necrosis factor family member ectodysplasin-A. J. Biol. Chem..

[90] Pääkkönen K (2001). The mutation spectrum of the EDA gene in X-linked anhidrotic ectodermal dysplasia. Hum. Mutat..

