# Supplementary material for: Refinement of evolutionary medicine predictions based on clinical evidence for the manifestations of Mendelian diseases
Source: Sci Rep. 2019 Dec 9;9:18577. doi: 10.1038/s41598-019-54976-4 (PMC6901466; doi:10.1038/s41598-019-54976-4)
Supplement: Supplementary file 1 — Supplementary Appendix 1 [file 41598_2019_54976_MOESM1_ESM.pdf]

Supplementary Appendix 1 for

Title: Refinement of evolutionary medicine predictions based on clinical evidence for the manifestations of Mendelian diseases

Authors: Daniela Šimčíková[1], Petr Heneberg[1]*.

Affiliation: [1]Charles University, Third Faculty of Medicine, Prague, Czech Republic.

*Correspondence to: Petr Heneberg, Third Faculty of Medicine, Charles University, Ruská 87, CZ-100 00 Prague, Czech Republic, Tel: ++420 – 775 311 177, Fax: ++420 – 267 162 710, E-mail: petr.heneberg@lf3.cuni.cz.

This file includes:

Supplementary Figure Legends, Supplementary Table Legends

Figures S1 to S5

SUPPLEMENTARY FIGURE LEGENDS

Fig. S1. Mean EVmutation scores (±SD) for disease-associated and no phenotype-associated variations. Horizontal lines illustrate the mean EVmutation scores for total disease-associated (-6.58) and no phenotype-associated (-3.86) variations.

Fig. S2. The implementation of MSA analyses based on the GV scores. (a) The relative percentages of variations with a non-zero GV score among the disease- and no phenotype-associated variations calculated for the 44 analyzed proteins. The lines indicate the relative percentages of variations with non-zero GV scores among total disease- and no phenotype-associated variations. (b) The EVmutation scores for disease-associated variations with non-zero GV scores. The lines indicate the median EVmutation scores for total disease-associated variations (lower line) and disease-associated variations with non-zero GV scores (upper line).

Fig. S3. The maximum likelihood phylogenetic trees of amino acid sequences predicted to be encoded by *AR* (a) and *PTEN* (b). Although amino acid sequences of the mammalian AR and PTEN proteins are highly conserved, the simple addition of several orthologs from more evolutionarily distant organisms increases the number of informative bases and thus enables the more efficient use of the MSA and GV approaches for inferring the evolutionary conservation of sites affected by missense variations. The bar indicates the number of substitutions per site. The branch lengths indicate the expected numbers of substitutions per amino acid site.

Fig. S4. The efficiency of the SNAP2 and PoPMuSiC 2.1 prediction methods in predicting the effects of missense variations with known clinical phenotypes on proteins that cause Mendelian diseases. (a-b) The distribution of the numerical SNAP2 (a) and PoPMuSiC 2.1 (b) scores calculated for missense variations with known clinical phenotypes. The vertical line indicates the arbitrary threshold for variations with an effect. (c-d) The relative percentage of correct predictions of disease and no clinical phenotypes by SNAP2 (c) and PoPMuSiC 2.1 (d) calculated for the 44 analyzed proteins.

Fig. S5. Scatter plots showing the EVmutation and SNAP2 scores for clinically observed disease- and no phenotype-associated variations (plots on the left side) and for the theoretical variations stratified into possible and impossible variations according to Bromberg *et al.*[15] (plots on the right side). (a-b) *AR*, (c-d) *ATP7A*, (e-f) *BMPR2*, (g-h) *BTK*, (i-j) *CD40LG*, (k-l) *CDKL5*, (m-n) *CPOX*, (o-p) *CYBB*, (q-r) *DCX*, (s-t) *DMD*, (u-v) *EDA*, (w-x) *ELANE*, (y-z) *F9*, (aa-ab) *FHL1*, (ac-ad) *FLNA*, (ae-af) *G6PD*, (ag-ah) *GCK*, (ai-aj) *GCH1*, (ak-al) *GLA*, (am-an) *HBB*, (ao-ap) *HDAC8*, (aq-ar) *HMBS*, (as-at) *HNF4A*, (au-av) *HPRT1*, (aw-ax) *HSPB1*, (ay-az) *IDS*, (ba-bb) *IL2RG*, (bc-bd) *ITGA2B*, (be-bf) *KIT*, (bg-bh) *MECP2*, (bi-bj) *MSH2*, (bk-bl) *OTC*, (bm-bn) *PDHA1*, (bo-bp) *PROC*, (bq-br) *PTEN*, (bs-bt) *PTPN11*, (bu-bv) *RET*, (bw-bx) *SERPING1*, (by-bz) *SH2D1A*, (ca-cb) *STK11*, (cc-cd) *TGFBR2*, (ce-cf) *TP63*, (cg-ch) *TTR*, and (ci-cj) *UROD*.

SUPPLEMENTARY TABLE LEGENDS

Table S1. Estimates of the evolutionary divergence among the indicated groups of vertebrates based on amino acid sequences predicted to be encoded by *AR*. The number of base differences per site calculated by averaging all sequence pairs between groups (distance) ± SE is shown.

Table S2. Estimates of the evolutionary divergence among the indicated groups of vertebrates based on amino acid sequences predicted to be encoded by *PTEN*. The number of base differences per site calculated by averaging all sequence pairs between groups (distance) ± SE is shown.

Table S3. The EVmutation and SNAP2 predictions for individual protein domains. The numbers of clinically observed disease- and no phenotype-associated variations, median values and SD of the predictions, differences in medians between the disease- and no phenotype-associated variations, and *P*-values resulting from *t*-tests with Bonferroni's correction that we used to determine the differences between the disease- and no phenotype-associated variations are shown.

Table S4. The EVmutation and SNAP2 scores for individual enzyme classes and proteins without enzymatic functions. The numbers of clinically observed disease- and no phenotype-associated variations, median values and SD of the predictions, differences in median values between the disease- and no phenotype-associated variations, and *p*-values resulting from Dunn's post-tests of Kruskal-Wallis one-way ANOVA on ranks are shown. The tested proteins included four classes of enzymes, oxidoreductases (EC1), transferases (EC2), hydrolases (EC3) and lyases (EC4), and proteins without enzymatic functions.

Table S5. The EVmutation, SNAP2 and PoPMuSiC 2.1 scores for diseases associated with the analyzed genes. The numbers of variations associated with the particular diseases and variations that are thought to be associated with no phenotypes are shown. Mean scores, SD, minimum and maximum scores are indicated. The data were tested for their normality using Shapiro-Wilk tests and then one-way ANOVA or Kruskal-Wallis one-way ANOVA on ranks was performed, and the resulting *F* or *H* and *p*-values are indicated. The presence of multiple phenotypes associated with each analyzed gene was analyzed using Dunn's or Tukey's post-tests, and their resulting *p*-values are indicated.

Table S6. The list of genes that were used to establish the model. The gene abbreviations, protein names, UniProt codes, ranges of amino acids covered by EVmutation, SNAP2 and PoPMuSiC 2.1 predictions, ranges of amino acids covered by all the three prediction methods, the identification numbers of structures in the PDB database, resolution of X-ray structures or the availability of the NMR structure, total length of the amino acid sequence of dominant isoform of the respective protein, NCBI accession numbers for the analyzed proteins and mRNAs, number of amino acids in Pfam domains and the names of these domains, Pfam domain abbreviations used in Fig. 2e, Pfam codes and EC numbers are indicated.

Table S7. The list of variations used to establish the model. The gene abbreviations, variations, codons encoding the original amino acids, classification of the possibility of variations according to Bromberg *et al.*,[15] EVmutation scores, SNAP2 scores, PoPMuSiC 2.1 scores, GV scores, GV scores based on the extended alignments of *AR* and *PTEN*, classifications of the variations, Pfam codes, EC codes, phenotypes, references used to classify the phenotypes, sources of the references, domains, classification of variations according to ClinVar, other sources used to classify variations, allele counts and numbers according to the ExAC browser, numbers of hemizygotes according to the ExAC browser, and

the numbers of disease-, no phenotype- and other phenotype-associated variations at the respective positions are indicated.

Table S8. The list of variations used to validate the model. The NCBI accession numbers, nucleotide substitutions, amino acid variations, original amino acids, positions of the amino acids, variant amino acids, gene abbreviations, clinical phenotypes (refer to as “Conditions”) according to ClinVar, clinical significance according to ClinVar, last revision dates according to ClinVar, review status according to ClinVar, the chromosome and location on the chromosome (releases GRCh37 and GRCh38), VariationID, AlleleID, EVmutation scores, SNAP2 scores, allele counts and numbers according to the ExAC browser, and the variation classification according to Sherloc are indicated.

Table S9. The list of genes used to validate the model. The associated disorders, inheritance modes, gene abbreviations, UniProt codes, protein lengths and NCBI accession numbers for the analyzed proteins are indicated.

Table S10. EVmutation and SNAP2 scores for the validation dataset. EVmutation and SNAP2 scores (means±SE) and the number of variations in each analyzed category are indicated. The categories are used as listed in ClinVar.

Table S11. Application of the American College of Medical Genetics and Genomics (ACMG) criteria for the classification of variations. The frequencies of disease-associated, partial phenotype-associated and no phenotype-associated variations that were stratified according to the ACMG criteria for the classification of variations into benign (1B, 3B and 5B) and pathogenic (0.5 P and 1P) according to the population frequencies of the variations are shown.

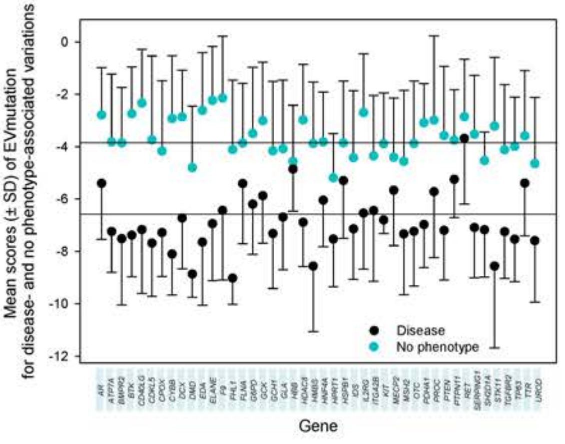
Mean scores (± SD) of EVmutation
for disease- and no phenotype-associated variations
0
-2
-4
-6
-8
-10
-12
Disease
No phenotype
AR
ATP7A
BMPR2
BTK
CD40LG
CDKL5
CPOX
CYBB
DCX
DMD
EDA
ELANE
F9
FHL1
FLNA
G6PD
GCK
GCH1
GLA
HBB
HDAC8
HMBS
HNF4A
HPRT1
HSPB1
IDS
IL2RG
ITGA2B
KIT
MECP2
MSH2
OTC
PDHA1
PROC
PTEN
PTPN11
RET
SERPING1
SH2D1A
STK11
TGFBR2
TP63
TTR
UROD
Gene

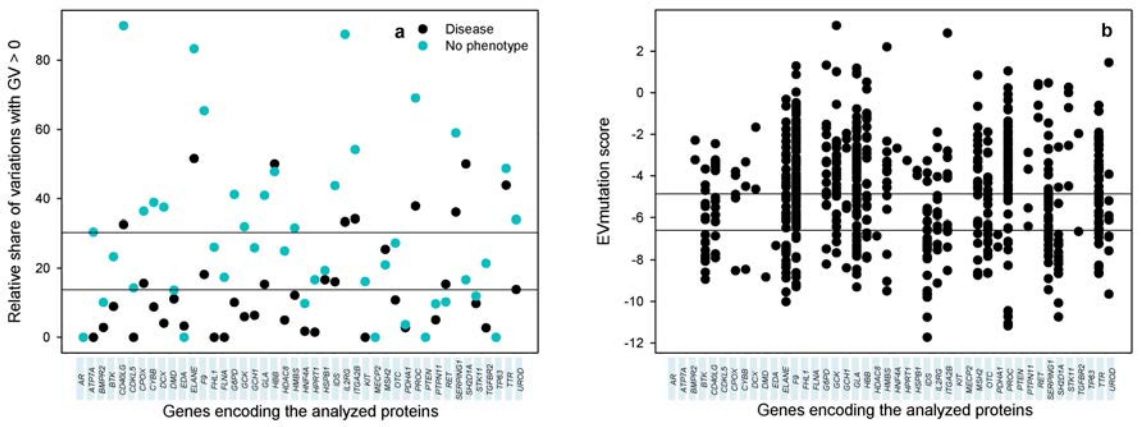
a
Disease
No phenotype
Relative share of variations with GV > 0
0
20
40
60
80
AR
ATP7A
BMPR2
BTK
CD40LG
CDKL5
CPOX
CYBB
DCX
DMD
EDA
ELANE
F9
FHL1
FLNA
G6PD
GCK
GCH1
GLA
HBB
HDAC8
HMBS
HNF4A
HPRT1
HSPB1
IDS
IL2RG
ITGA2B
KIT
MECP2
MSH2
OTC
PDHA1
PROC
PTEN
PTPN11
RET
SERPING1
SH2D1A
STK11
TGFBR2
TP63
TTR
UROD
Genes encoding the analyzed proteins
b
EVmutation score
2
0
-2
-4
-6
-8
-10
-12
AR
ATP7A
BMPR2
BTK
CD40LG
CDKL5
CPOX
CYBB
DCX
DMD
EDA
ELANE
F9
FHL1
FLNA
G6PD
GCK
GCH1
GLA
HBB
HDAC8
HMBS
HNF4A
HPRT1
HSPB1
IDS
IL2RG
ITGA2B
KIT
MECP2
MSH2
OTC
PDHA1
PROC
PTEN
PTPN11
RET
SERPING1
SH2D1A
STK11
TGFBR2
TP63
TTR
UROD
Genes encoding the analyzed proteins

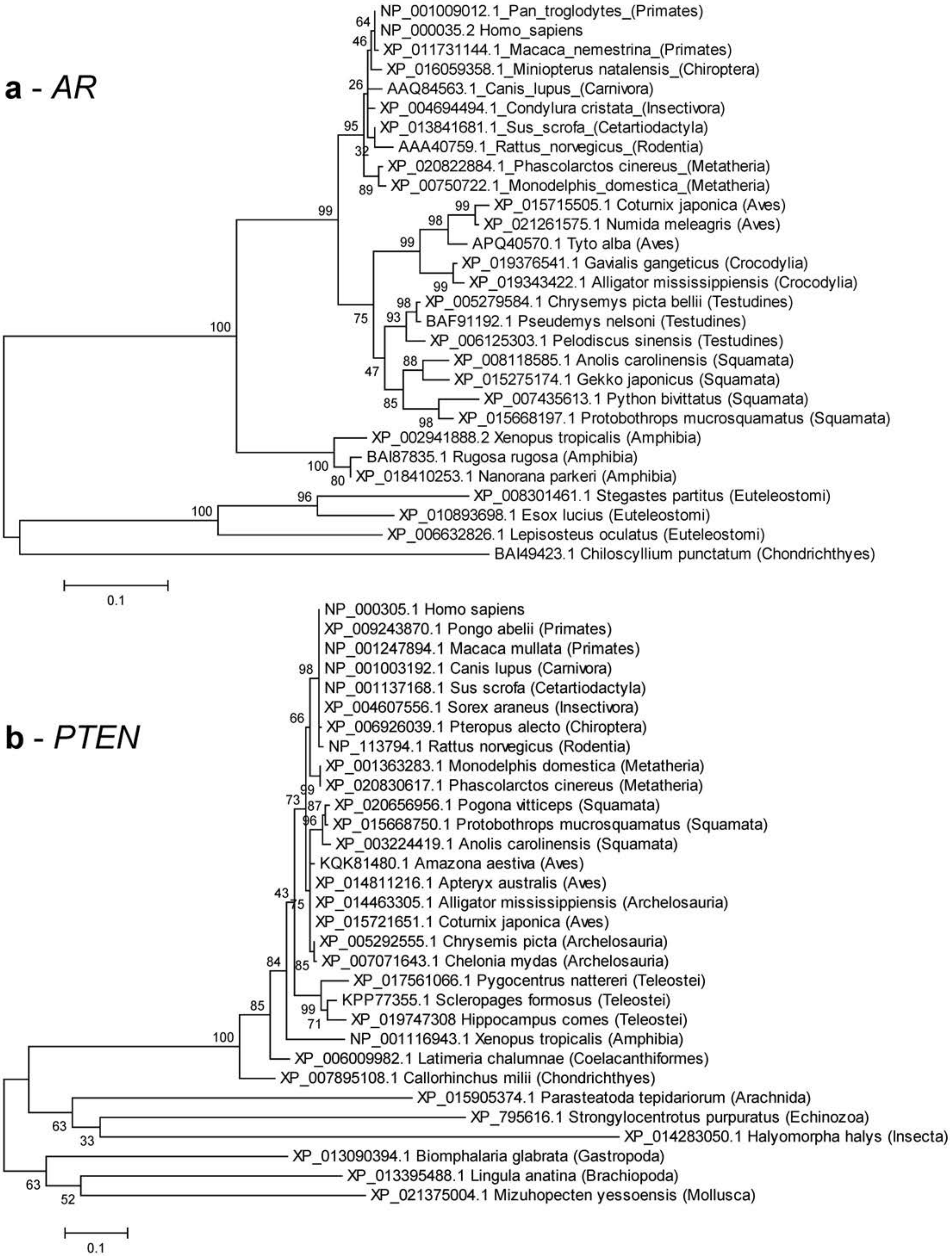

a - AR
NP_001009012.1_Pan_troglodytes_(Primates)
NP_000035.2 Homo_sapiens
XP_011731144.1_Macaca_nemestrina_(Primates)
XP_016059358.1_Miniopterus natalensis_(Chiroptera)
AAQ84563.1_Canis_lupus_(Carnivora)
XP_004694494.1_Condylura cristata_(Insectivora)
XP_013841681.1_Sus_scrofa_(Cetartiodactyla)
AAA40759.1_Rattus_norvegicus_(Rodentia)
XP_020822884.1_Phascolarctos cinereus_(Metatheria)
XP_00750722.1_Monodelphis_domestica_(Metatheria)
XP_015715505.1 Coturnix japonica (Aves)
XP_021261575.1 Numida meleagris (Aves)
APQ40570.1 Tyto alba (Aves)
XP_019376541.1 Gavialis gangeticus (Crocodylia)
XP_019343422.1 Alligator mississippiensis (Crocodylia)
XP_005279584.1 Chrysemys picta bellii (Testudines)
BAF91192.1 Pseudemys nelsoni (Testudines)
XP_006125303.1 Pelodiscus sinensis (Testudines)
XP_008118585.1 Anolis carolinensis (Squamata)
XP_015275174.1 Gekko japonicus (Squamata)
XP_007435613.1 Python bivittatus (Squamata)
XP_015668197.1 Protobothrops mucrosquamatus (Squamata)
XP_002941888.2 Xenopus tropicalis (Amphibia)
BAI87835.1 Rugosa rugosa (Amphibia)
XP_018410253.1 Nanorana parkeri (Amphibia)
XP_008301461.1 Stegastes partitus (Euteleostomi)
XP_010893698.1 Esox lucius (Euteleostomi)
XP_006632826.1 Lepisosteus oculatus (Euteleostomi)
BAI49423.1 Chiloscyllium punctatum (Chondrichthyes)
64
46
26
95
32
89
99
99
98
99
99
98
93
75
88
47
85
98
100
100
80
100
96
100
0.1
b - PTEN
NP_000305.1 Homo sapiens
XP_009243870.1 Pongo abelii (Primates)
NP_001247894.1 Macaca mullata (Primates)
NP_001003192.1 Canis lupus (Carnivora)
NP_001137168.1 Sus scrofa (Cetartiodactyla)
XP_004607556.1 Sorex araneus (Insectivora)
XP_006926039.1 Pteropus alecto (Chiroptera)
NP_113794.1 Rattus norvegicus (Rodentia)
XP_001363283.1 Monodelphis domestica (Metatheria)
XP_020830617.1 Phascolarctos cinereus (Metatheria)
XP_020656956.1 Pogona vitticeps (Squamata)
XP_015668750.1 Protobothrops mucrosquamatus (Squamata)
XP_003224419.1 Anolis carolinensis (Squamata)
KQK81480.1 Amazona aestiva (Aves)
XP_014811216.1 Apteryx australis (Aves)
XP_014463305.1 Alligator mississippiensis (Archelosauria)
XP_015721651.1 Coturnix japonica (Aves)
XP_005292555.1 Chrysemis picta (Archelosauria)
XP_007071643.1 Chelonia mydas (Archelosauria)
XP_017561066.1 Pygocentrus nattereri (Teleostei)
KPP77355.1 Scleropages formosus (Teleostei)
XP_019747308 Hippocampus comes (Teleostei)
NP_001116943.1 Xenopus tropicalis (Amphibia)
XP_006009982.1 Latimeria chalumnae (Coelacanthiformes)
XP_007895108.1 Callorhinchus milii (Chondrichthyes)
XP_015905374.1 Parasteatoda tepidariorum (Arachnida)
XP_795616.1 Strongylocentrotus purpuratus (Echinozoa)
XP_014283050.1 Halyomorpha halys (Insecta)
XP_013090394.1 Biomphalaria glabrata (Gastropoda)
XP_013395488.1 Lingula anatina (Brachiopoda)
XP_021375004.1 Mizuhopecten yessoensis (Mollusca)
98
66
99
73
87
96
43
75
85
84
85
99
71
100
63
33
63
52
0.1

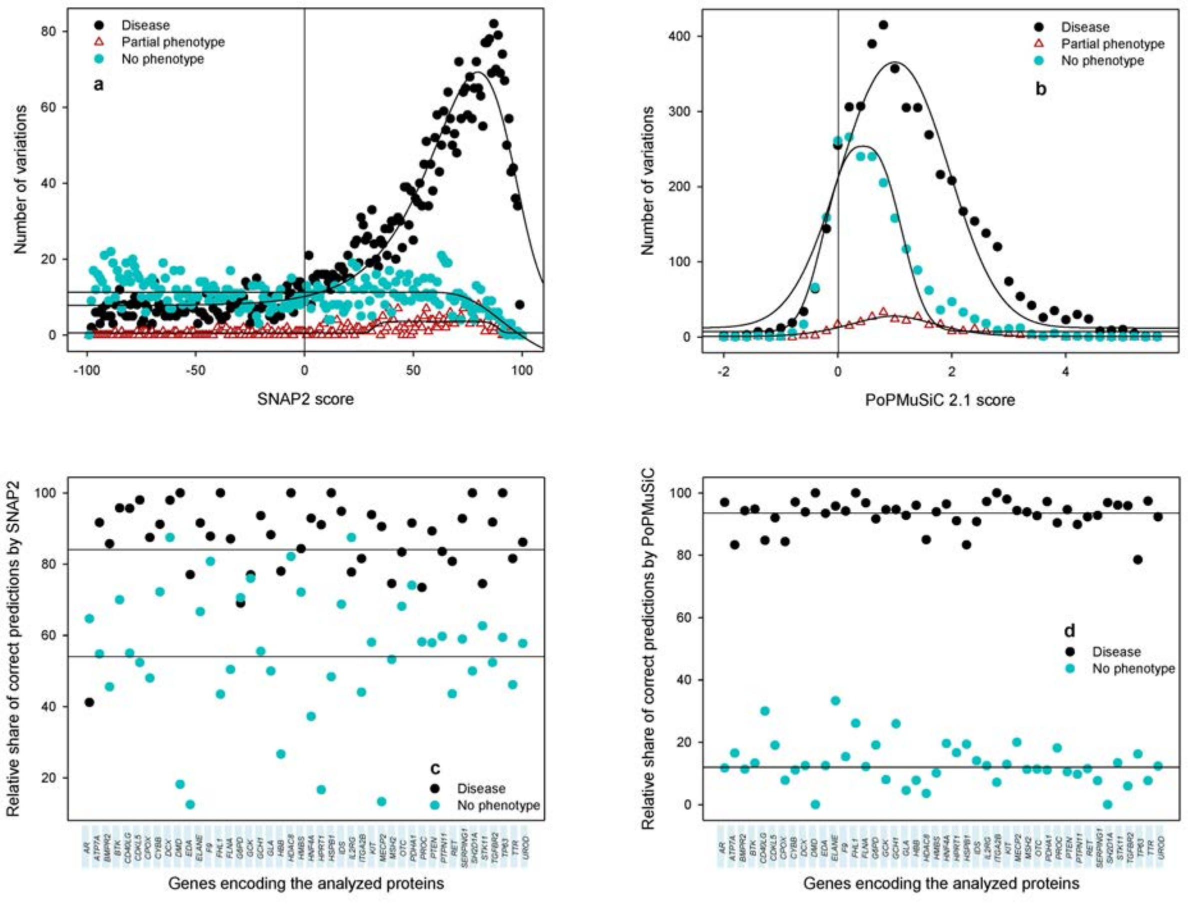

Disease
Partial phenotype
No phenotype
a
Number of variations
SNAP2 score
-100
-50
0
50
100
20
40
60
80
Disease
Partial phenotype
No phenotype
b
Number of variations
PoPMuSiC 2.1 score
-2
0
2
4
100
200
300
400
c
Disease
No phenotype
Relative share of correct predictions by SNAP2
Genes encoding the analyzed proteins
AR ATP7A BMPR2 BTK CD40LG CDKL5 CPOX CYBB DCX DMD EDA ELANE F9 FHL1 FLNA G6PD GCK GCH1 GLA HBB HDAC8 HMBS HNF4A HPRT1 HSPB1 IDS IL2RG ITGA2B KIT MECP2 MSH2 OTC PDHA1 PROC PTEN PTPN11 RET SERPING1 SH2D1A STK11 TGFBR2 TP63 TTR UROD
d
Disease
No phenotype
Relative share of correct predictions by PoPMuSiC
Genes encoding the analyzed proteins
0
20
40
60
80
100

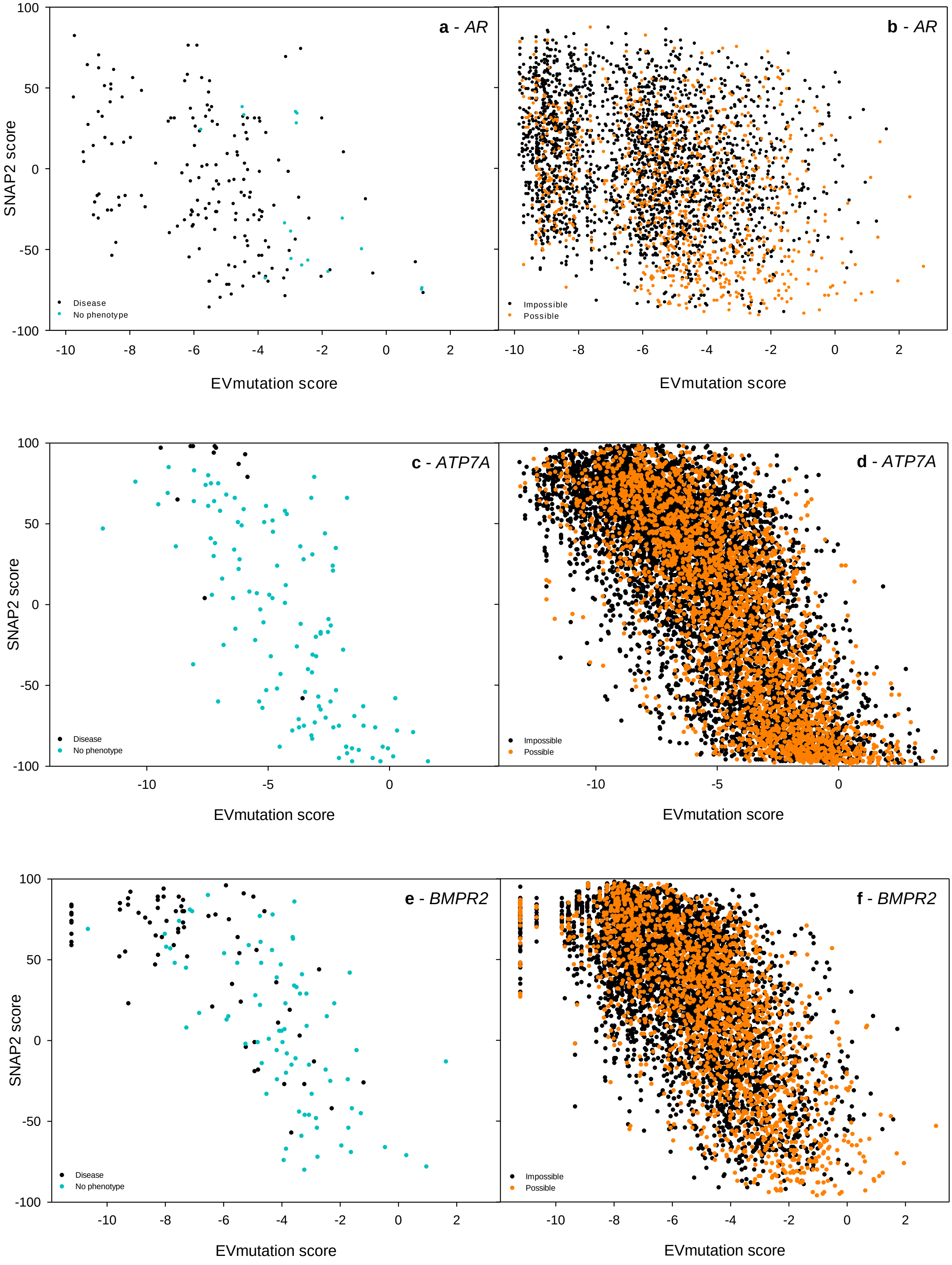
a - AR
b - AR
c - ATP7A
d - ATP7A
e - BMPR2
f - BMPR2
SNAP2 score
EVmutation score
Disease
No phenotype
Impossible
Possible
100
50
0
-50
-100
-10
-8
-6
-4
-2
0
2
-5

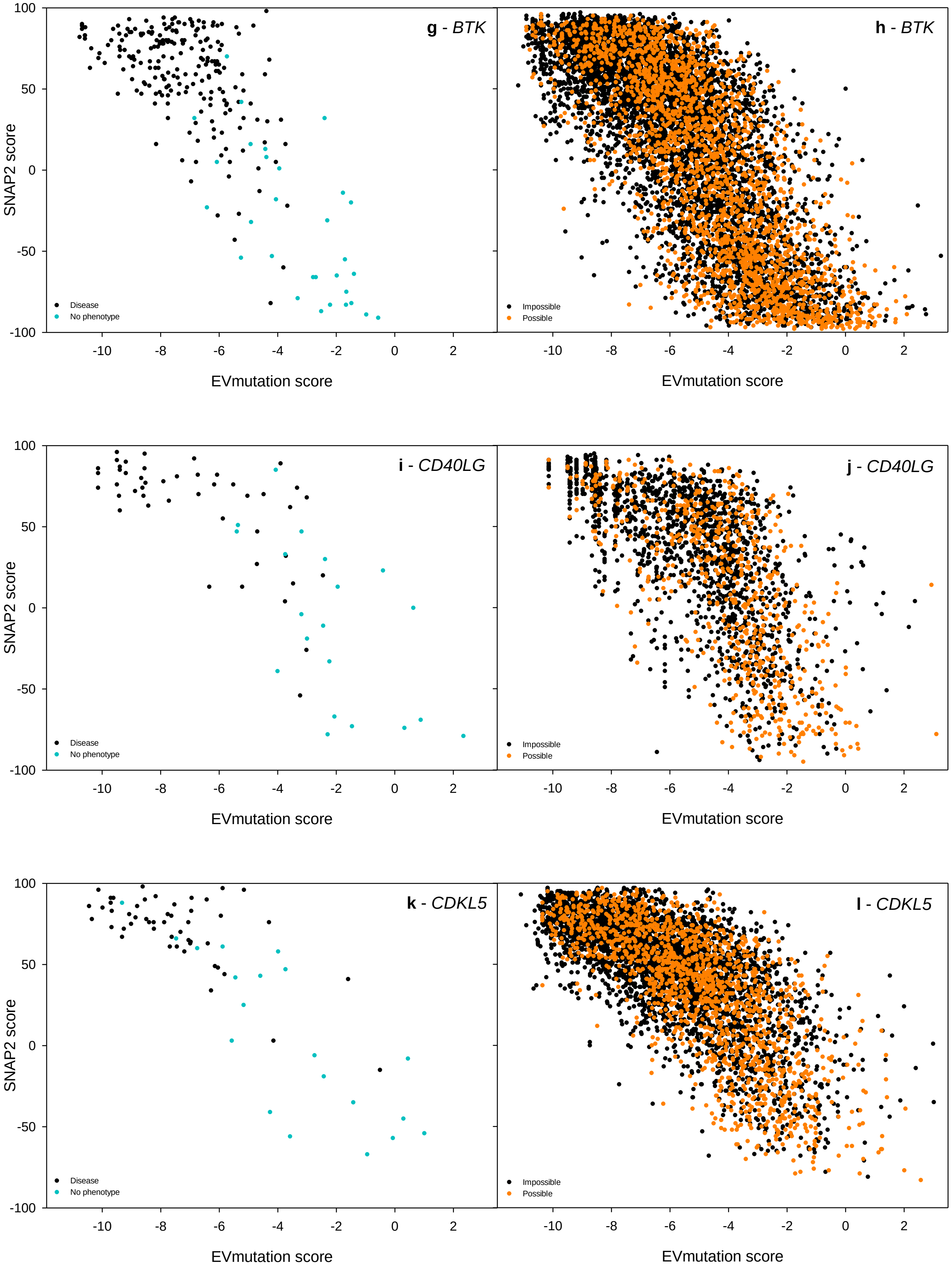

g - BTK
h - BTK
i - CD40LG
j - CD40LG
k - CDKL5
l - CDKL5
SNAP2 score
EVmutation score
Disease
No phenotype
Impossible
Possible
100
50
0
-50
-100
-10
-8
-6
-4
-2
0
2

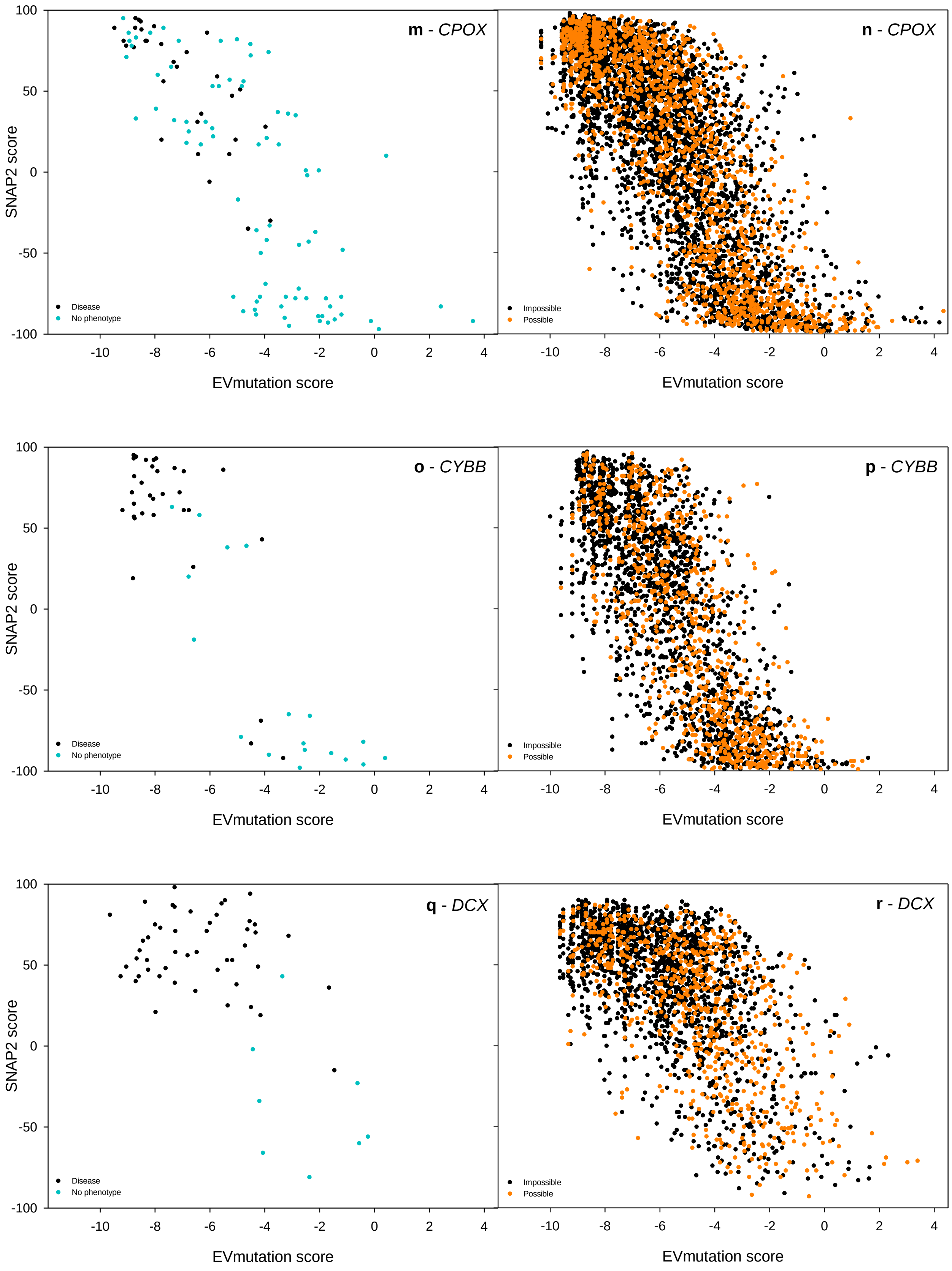

m - CPOX
n - CPOX
o - CYBB
p - CYBB
q - DCX
r - DCX
SNAP2 score
EVmutation score
100
50
0
-50
-100
-10
-8
-6
-4
-2
0
2
4
Disease
No phenotype
Impossible
Possible

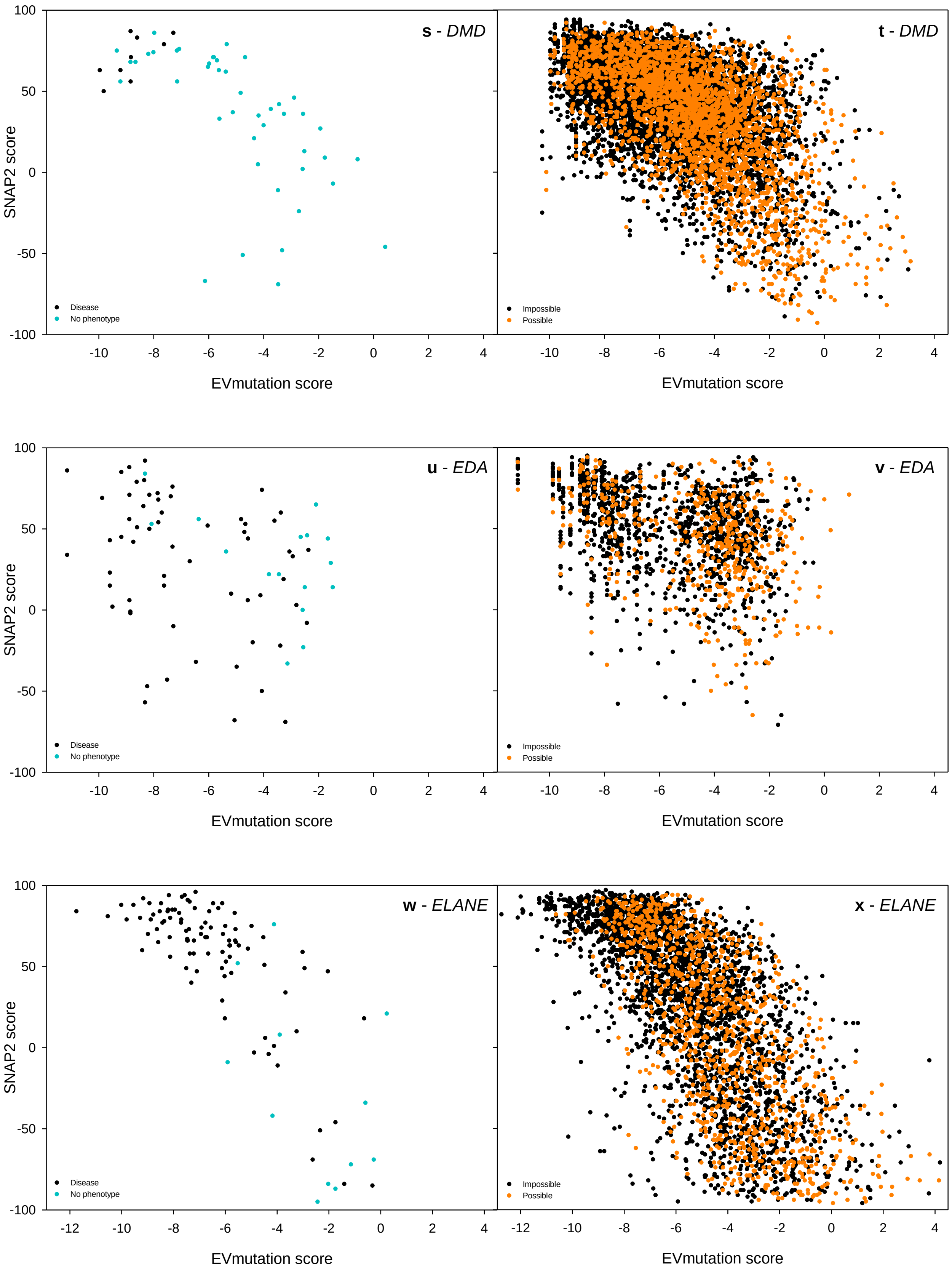

s - DMD
t - DMD
u - EDA
v - EDA
w - ELANE
x - ELANE
SNAP2 score
EVmutation score
Disease
No phenotype
Impossible
Possible
100
50
0
-50
-100
-12
-10
-8
-6
-4
-2
0
2
4

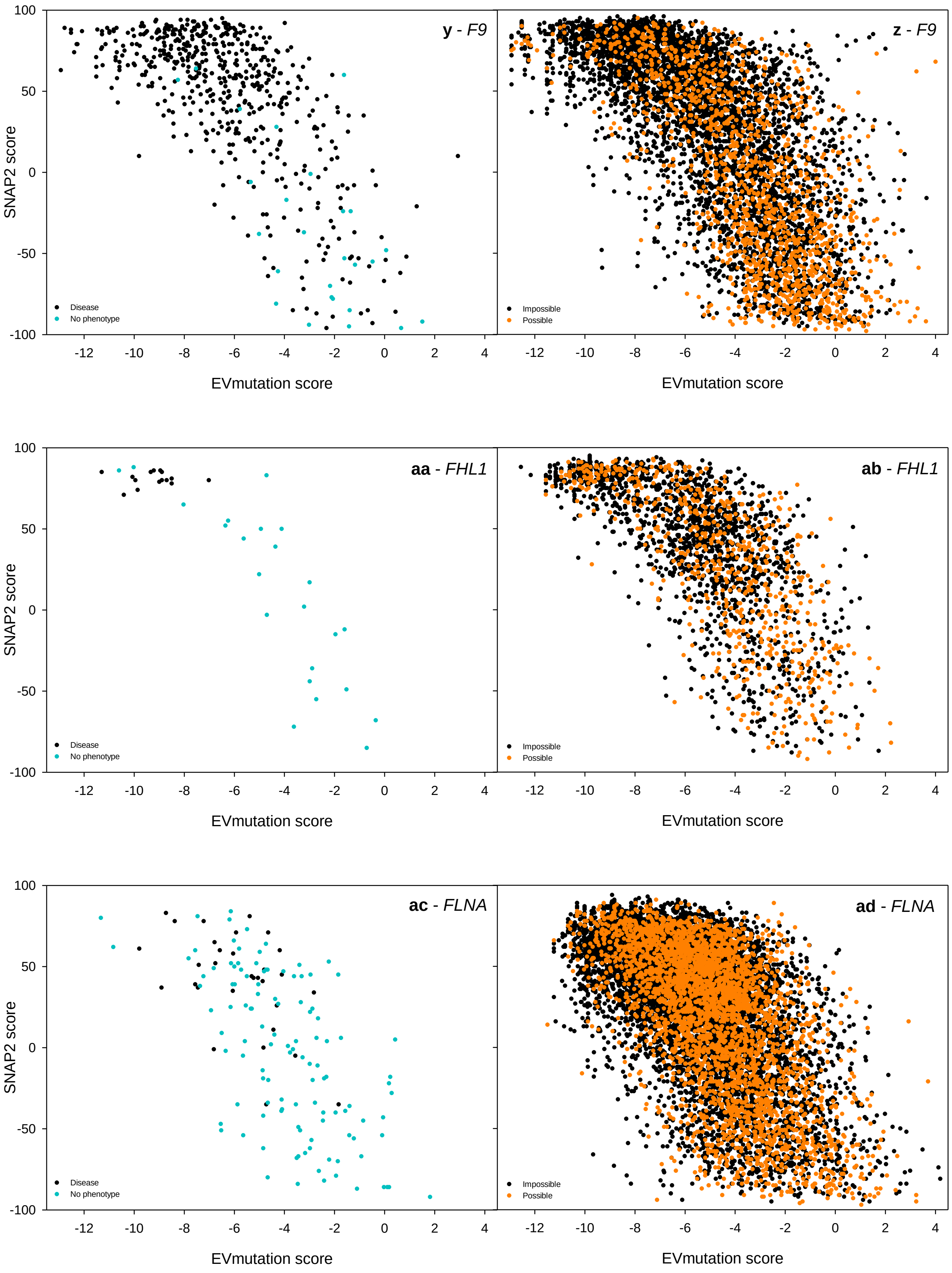

y - F9
z - F9
aa - FHL1
ab - FHL1
ac - FLNA
ad - FLNA
SNAP2 score
EVmutation score
Disease
No phenotype
Impossible
Possible
100
50
0
-50
-100
-12
-10
-8
-6
-4
-2
0
2
4

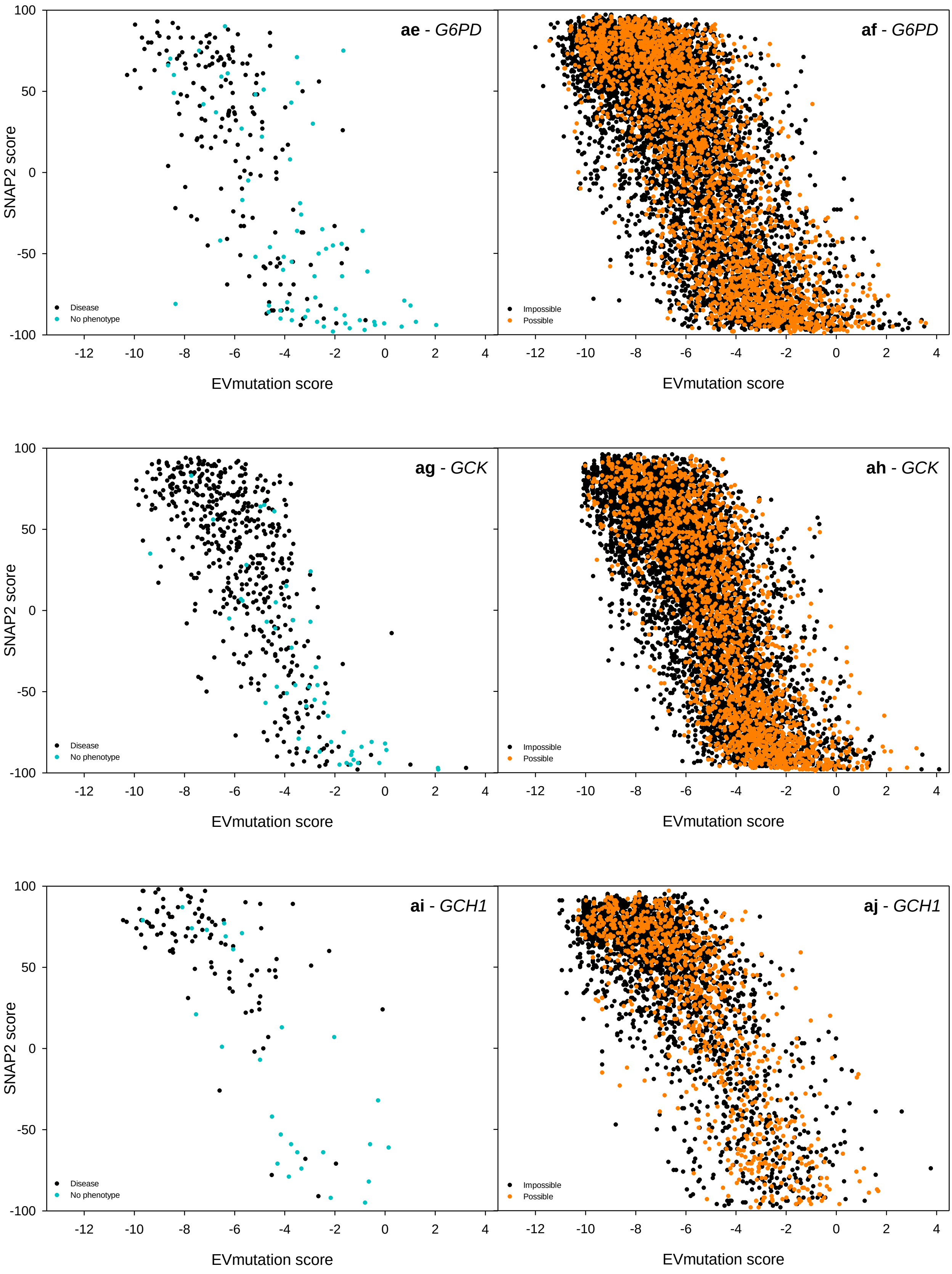

ae - G6PD
af - G6PD
ag - GCK
ah - GCK
ai - GCH1
aj - GCH1
SNAP2 score
EVmutation score
Disease
No phenotype
Impossible
Possible
100
50
0
-50
-100
-12
-10
-8
-6
-4
-2
0
2
4

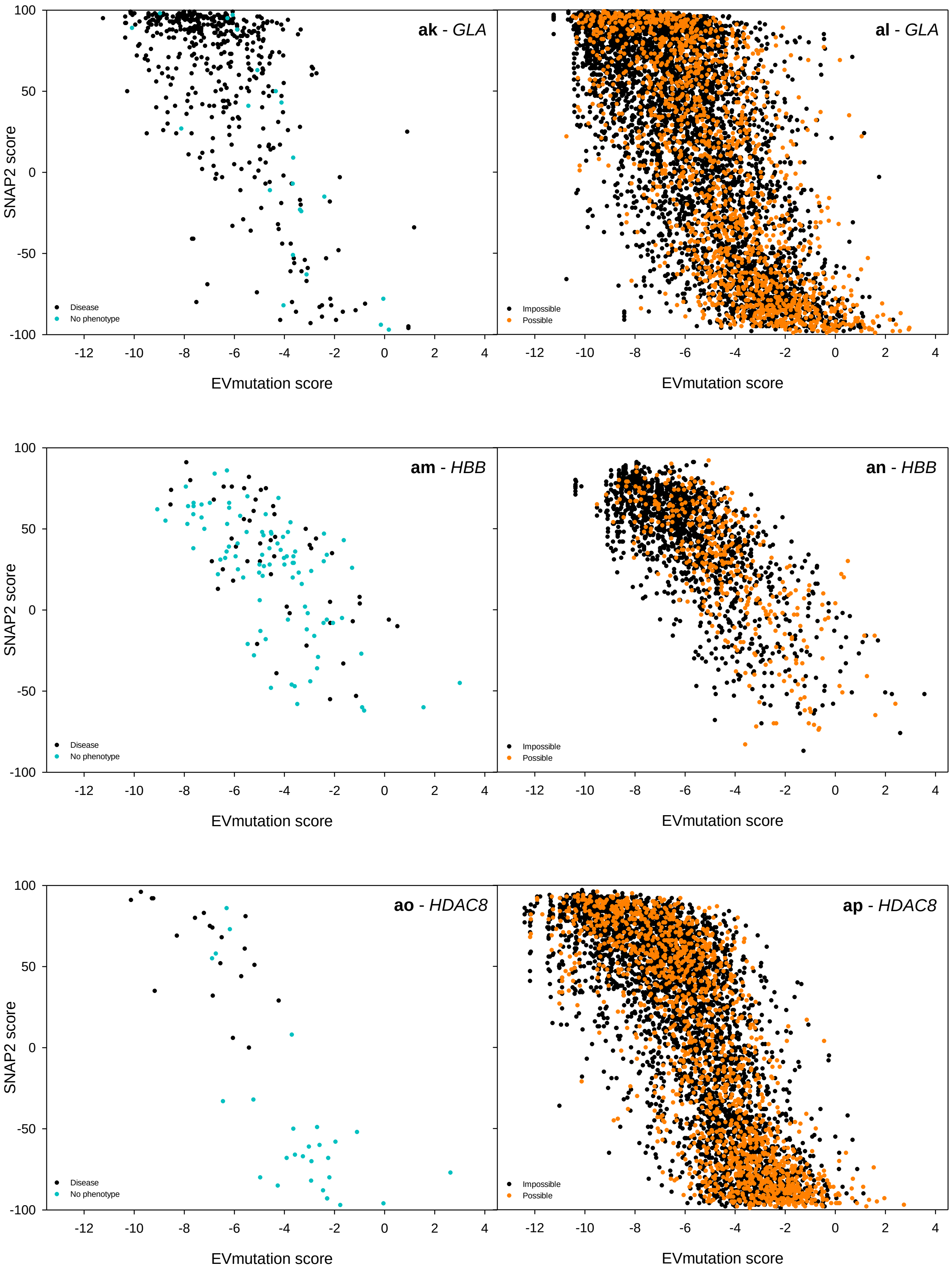
ak - GLA
al - GLA
am - HBB
an - HBB
ao - HDAC8
ap - HDAC8
SNAP2 score
EVmutation score
Disease
No phenotype
Impossible
Possible
100
50
0
-50
-100
-12
-10
-8
-6
-4
-2
0
2
4

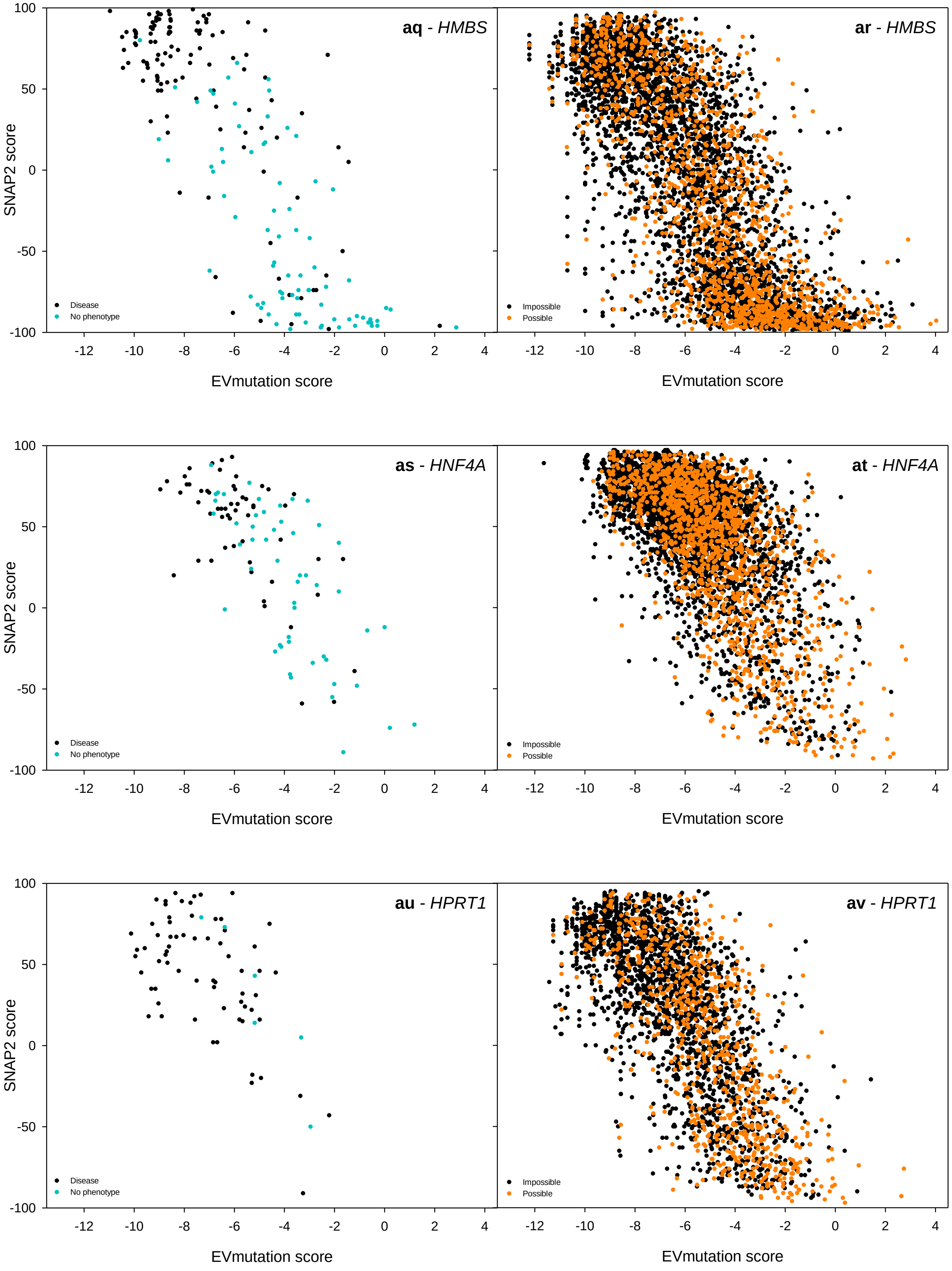
aq - HMBS
ar - HMBS
as - HNF4A
at - HNF4A
au - HPRT1
av - HPRT1
SNAP2 score
EVmutation score
100
50
0
-50
-100
-12
-10
-8
-6
-4
-2
0
2
4
Disease
No phenotype
Impossible
Possible

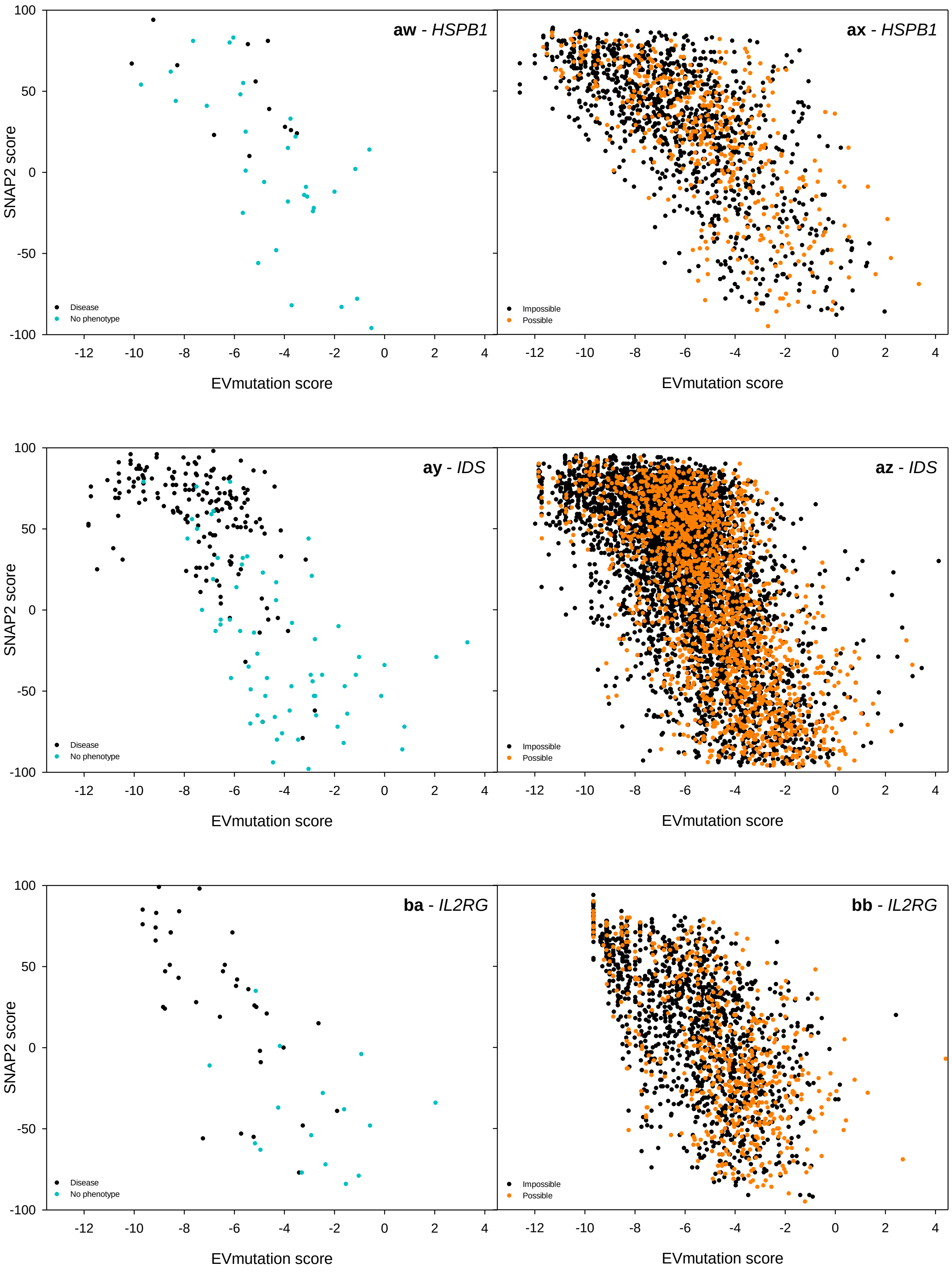

aw - HSPB1
ax - HSPB1
ay - IDS
az - IDS
ba - IL2RG
bb - IL2RG
SNAP2 score
EVmutation score
Disease
No phenotype
Impossible
Possible

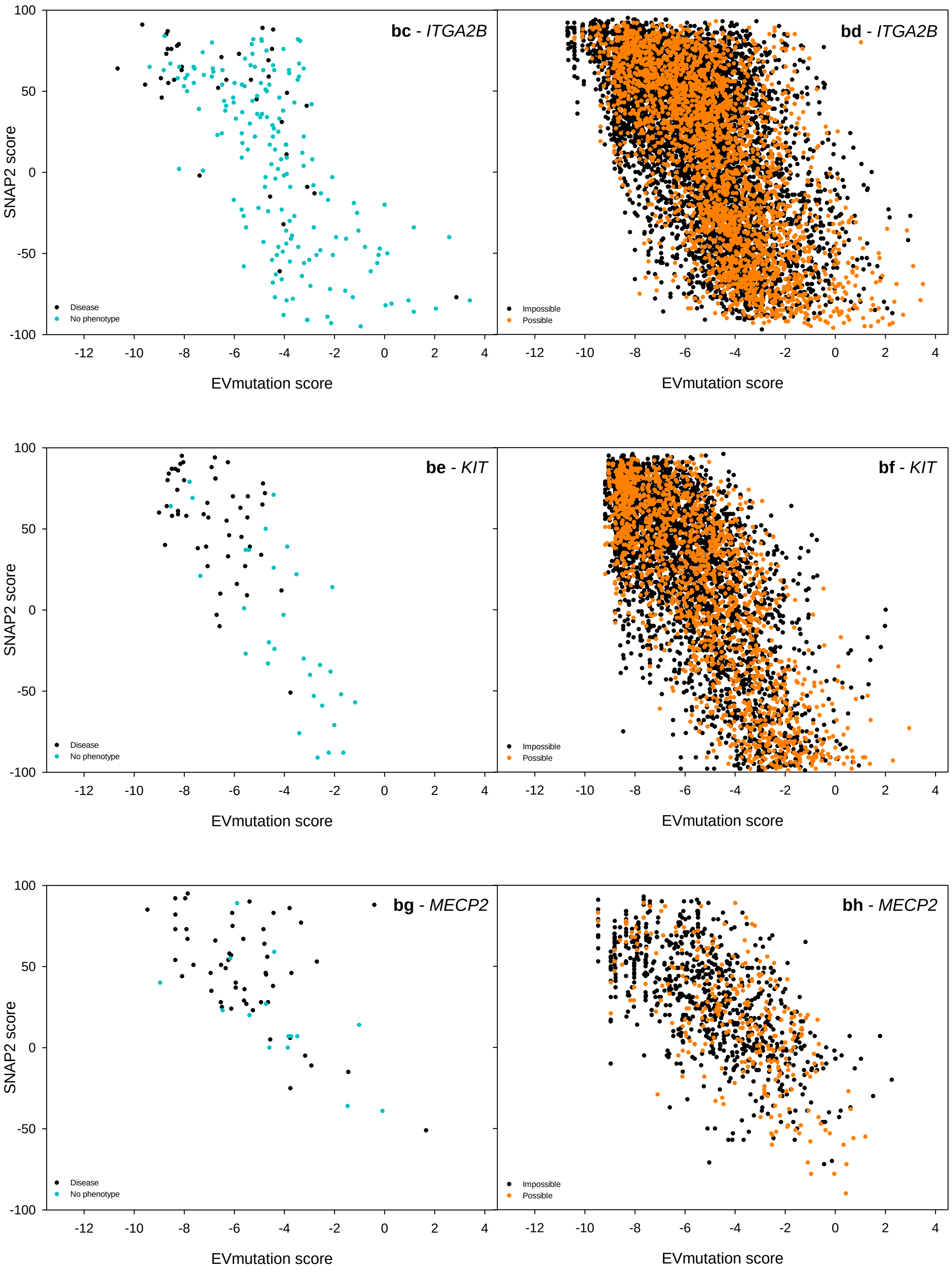

bc - ITGA2B
bd - ITGA2B
be - KIT
bf - KIT
bg - MECP2
bh - MECP2
SNAP2 score
EVmutation score
Disease
No phenotype
Impossible
Possible
100
50
0
-50
-100
-12
-10
-8
-6
-4
-2
0
2
4

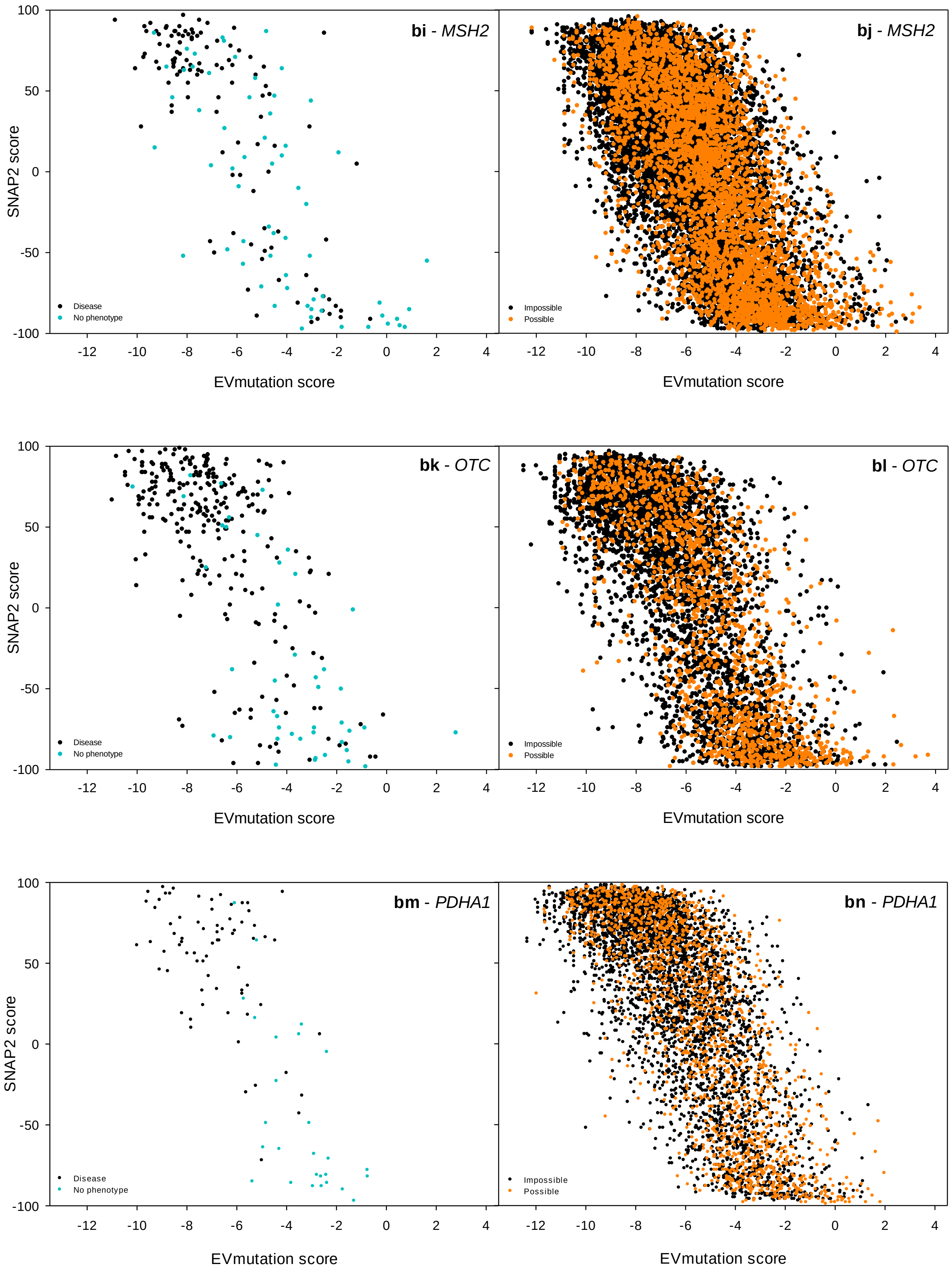

bi - MSH2
bj - MSH2
bk - OTC
bl - OTC
bm - PDHA1
bn - PDHA1
SNAP2 score
EVmutation score
100
50
0
-50
-100
-12
-10
-8
-6
-4
-2
0
2
4
Disease
No phenotype
Impossible
Possible

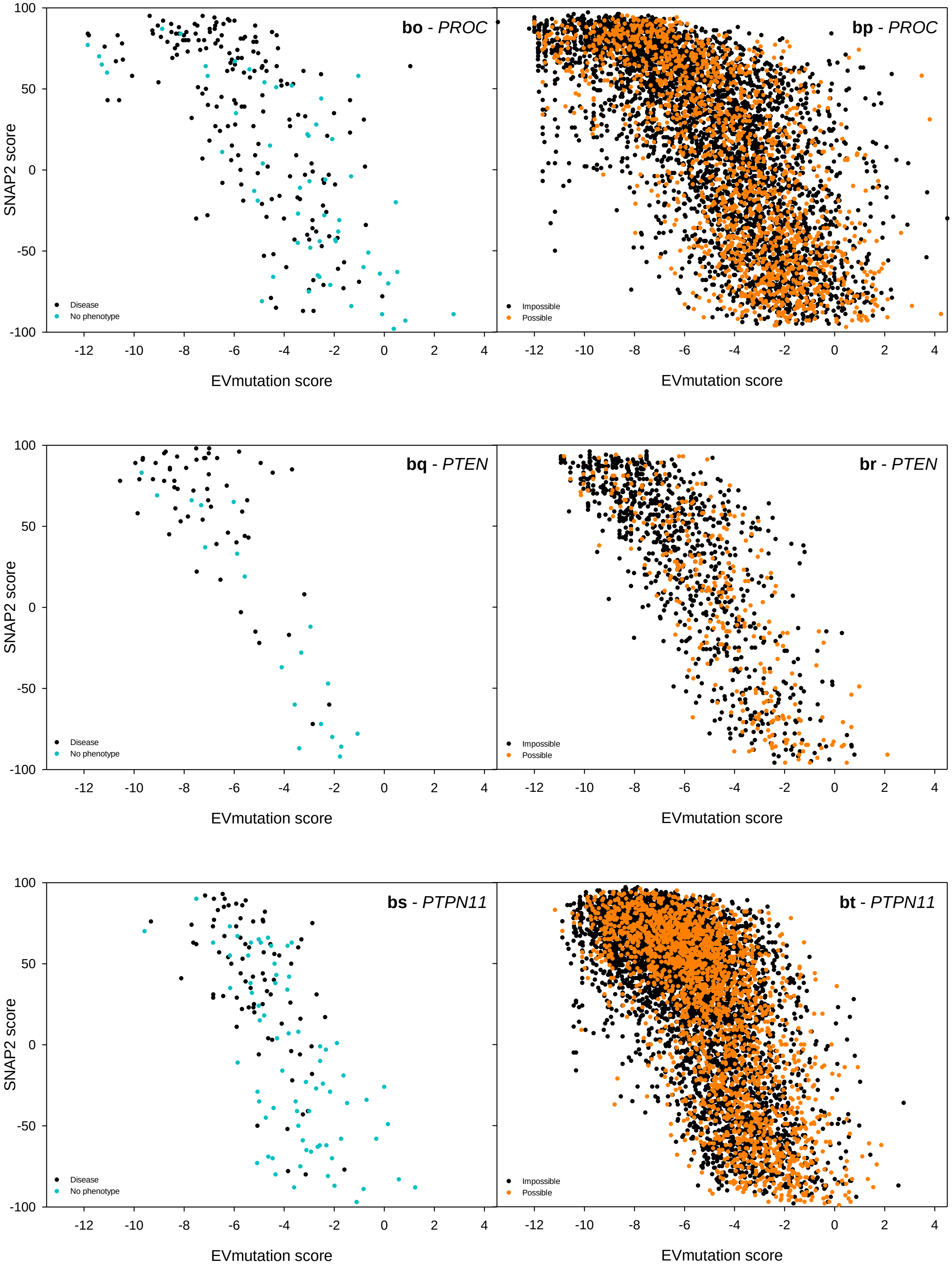

bo - PROC
bp - PROC
bq - PTEN
br - PTEN
bs - PTPN11
bt - PTPN11
SNAP2 score
EVmutation score
Disease
No phenotype
Impossible
Possible
100
50
0
-50
-100
-12
-10
-8
-6
-4
-2
0
2
4

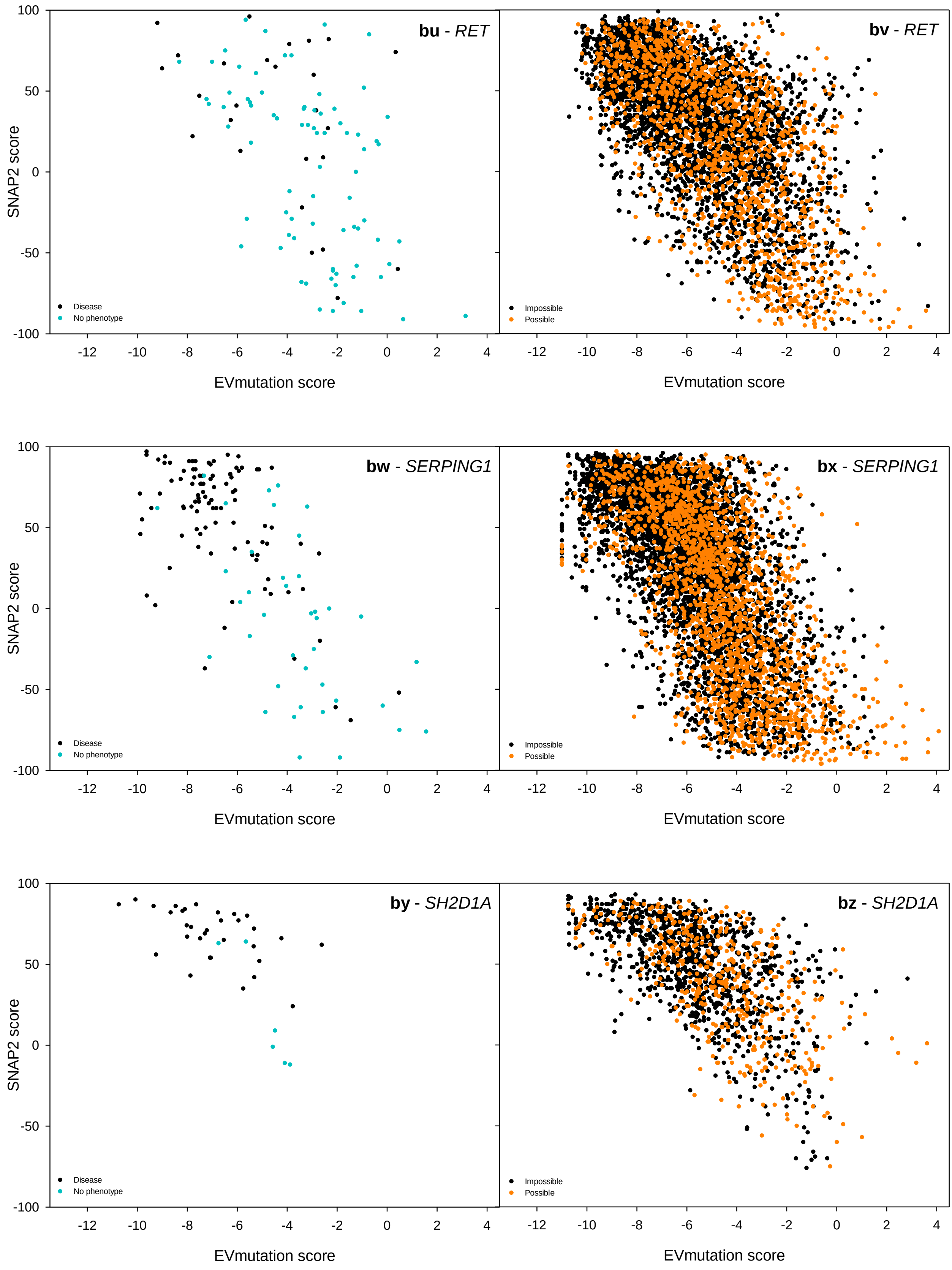
bu - RET
bv - RET
bw - SERPING1
bx - SERPING1
by - SH2D1A
bz - SH2D1A
SNAP2 score
EVmutation score
Disease
No phenotype
Impossible
Possible
100
50
0
-50
-100
-12
-10
-8
-6
-4
-2
0
2
4

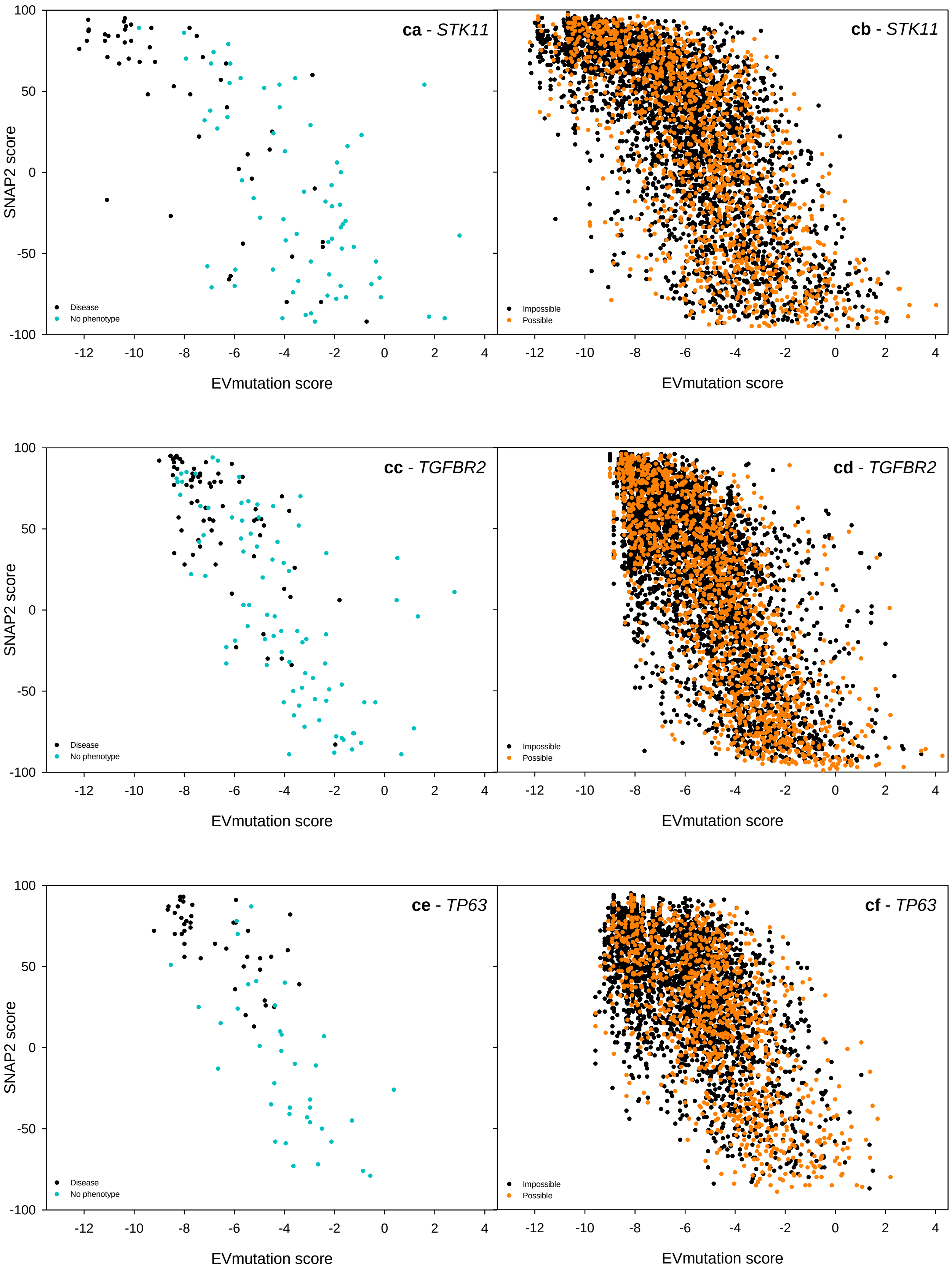
ca - STK11
cb - STK11
cc - TGFBR2
cd - TGFBR2
ce - TP63
cf - TP63
Disease
No phenotype
Impossible
Possible
SNAP2 score
EVmutation score
100
50
0
-50
-100
-12
-10
-8
-6
-4
-2
0
2
4

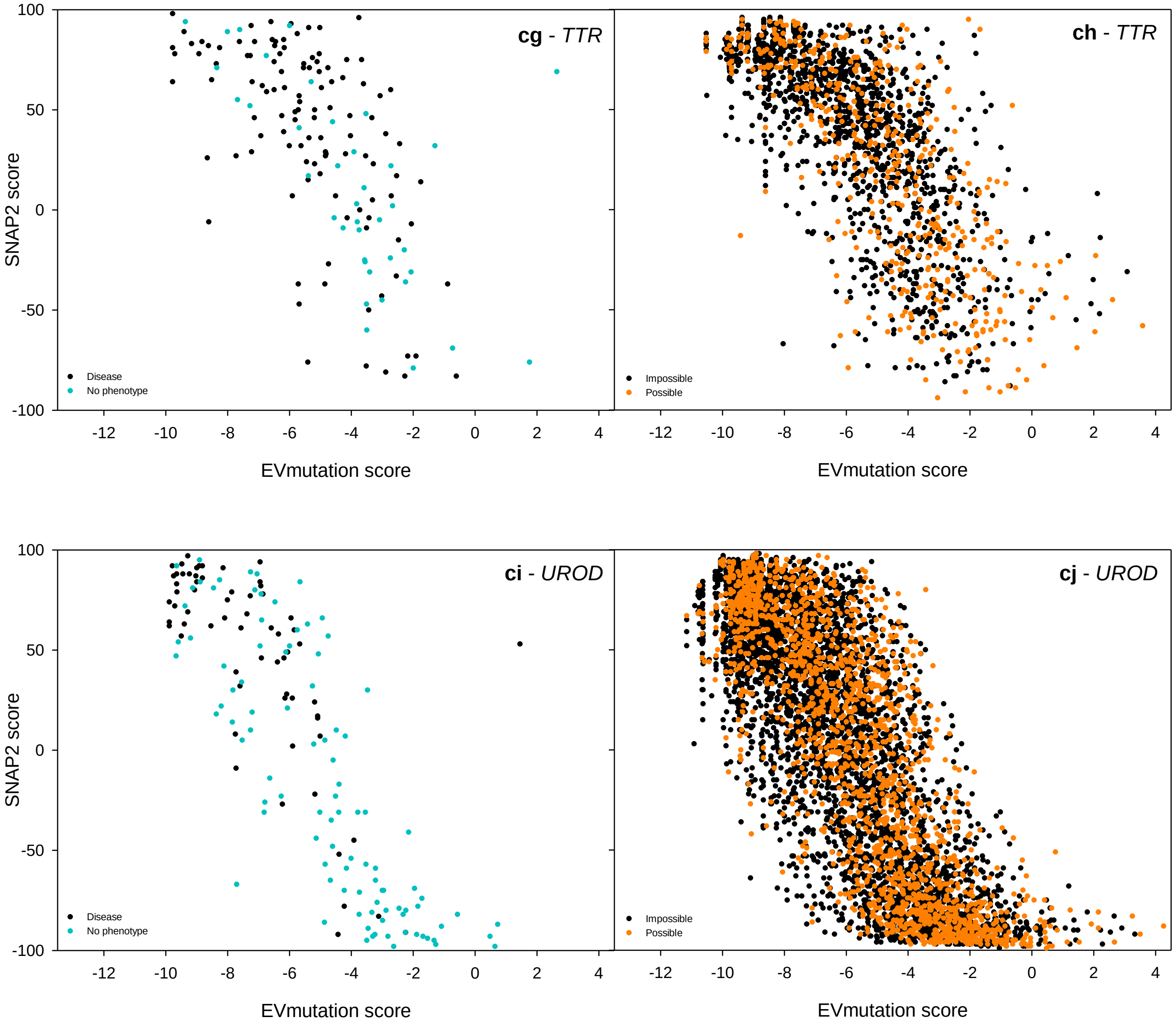

cg - *TTR*
ch - *TTR*
ci - *UROD*
cj - *UROD*
SNAP2 score
EVmutation score
100
50
0
-50
-100
-12
-10
-8
-6
-4
-2
0
2
4
Disease
No phenotype
Impossible
Possible
